# Prophylactic administration of probiotic strains *Weizmannia coagulans* and *Saccharomyces boulardii* attenuates early dysplastic changes in an AOM/DSS-induced mouse model

**DOI:** 10.1186/s12866-026-05634-z

**Published:** 2026-10-06

**Authors:** Marwa Matboli, Sanaa Eissa, Reham Hussein Mohamed, Nada Kotb, Ghada Galal Hamam, Mai Tarek, Nagwa M. Abo El Magd, Abdullah M. Abdo, Aisha Mohamed Hussein, Naydeen Mostafa, Marwa Mostafa Kamel

**Affiliations:** 1 Medical Biochemistry and Molecular Biology Department, Faculty of Medicine, Ain Shams University, P.O. box 11381, Abbassia, Cairo, Egypt; 2 Translational & Applied Science Hub, Faculty of Medicine, Ain Shams University, P.O. box 11381, Abbassia, Cairo, Egypt; 3 MASRI, Faculty of Medicine, Ain Shams University Research Center, Cairo, Egypt; 4 Clinical Pharmacology Department, Faculty of Medicine, Ain Shams University, Cairo, Egypt; 5 Department of Medical Histology, Faculty of Medicine, Ain Shams University, Cairo, Egypt; 6 Department of Medical Microbiology and Immunology, Faculty of Medicine, Ain Shams University, Cairo, Egypt; 7 Botany and Microbiology Department, Faculty of Science, Al-Azhar University, P.O. Box 11884, Nasr City, Cairo, Egypt; 8 Biochemistry and Molecular Biology Department, Oncology Diagnostic Research Unit (ODU), Faculty of Medicine, Ain Shams University, Cairo, Egypt; 9 Faculty of Medicine, Ain Shams University, Cairo, Egypt

**Keywords:** Probiotics, *Weizmannia coagulans*, *Saccharomyces boulardii*, AOM/DSS mouse model, Inflammation, miRNAs, Colorectal dysplasia, qRT-PCR

## Abstract

**Background:**

Colorectal dysplasia represents an early stage of colorectal carcinogenesis and is closely associated with chronic inflammation and altered microRNA (miRNA) expression. Probiotic strains such as *Weizmannia coagulans (formerly Bacillus coagulans)* and *Saccharomyces boulardii* possess anti-inflammatory properties and may influence host miRNA expression, suggesting their potential as prophylactic agents against inflammation-associated colorectal dysplasia.

**Methods:**

Mice received prophylactic oral administration of *Weizmannia coagulans* or *Saccharomyces boulardii* beginning one week before azoxymethane/dextran sulfate sodium (AOM/DSS) induction and continuing throughout the experimental period. Histopathological examination, TNF-α immunohistochemistry, serum pro-inflammatory cytokine measurements, and miRNA expression profiling using reverse transcription quantitative polymerase chain reaction (RT-qPCR) were performed to evaluate the prophylactic effects of both probiotic strains.

**Results:**

Prophylactic administration of both probiotic strains significantly attenuated early dysplastic alterations and inflammatory cell infiltration compared with the AOM/DSS group (*p* < 0.05). These histopathological improvements were accompanied by significantly lower serum levels of the pro-inflammatory cytokines IL-6 and IL-1β, together with reduced TNF-α immunoreactivity (*p* < 0.05). In addition, probiotic pretreatment attenuated the altered miRNA expression profile induced by AOM/DSS. Compared with the model group, expression of miR-122-5p, miR-29b-5p, and miR-15b-5p was significantly reduced, whereas miR-98-5p, miR-202-5p, miR-21-5p, and let-7i-5p showed increased expression and were associated with miRNA expression patterns that more closely resembled those of the control group (*p* < 0.01).

**Conclusion:**

Prophylactic administration of *Weizmannia coagulans* and *Saccharomyces boulardii* reduced inflammation-associated early dysplastic changes while preserving normal miRNA expression patterns in an AOM/DSS-induced murine model. These findings support the potential of probiotic prophylaxis as an early preventive strategy against colorectal dysplasia and provide a foundation for future studies investigating its clinical applicability.

**Graphical Abstract:**

**Figure:**
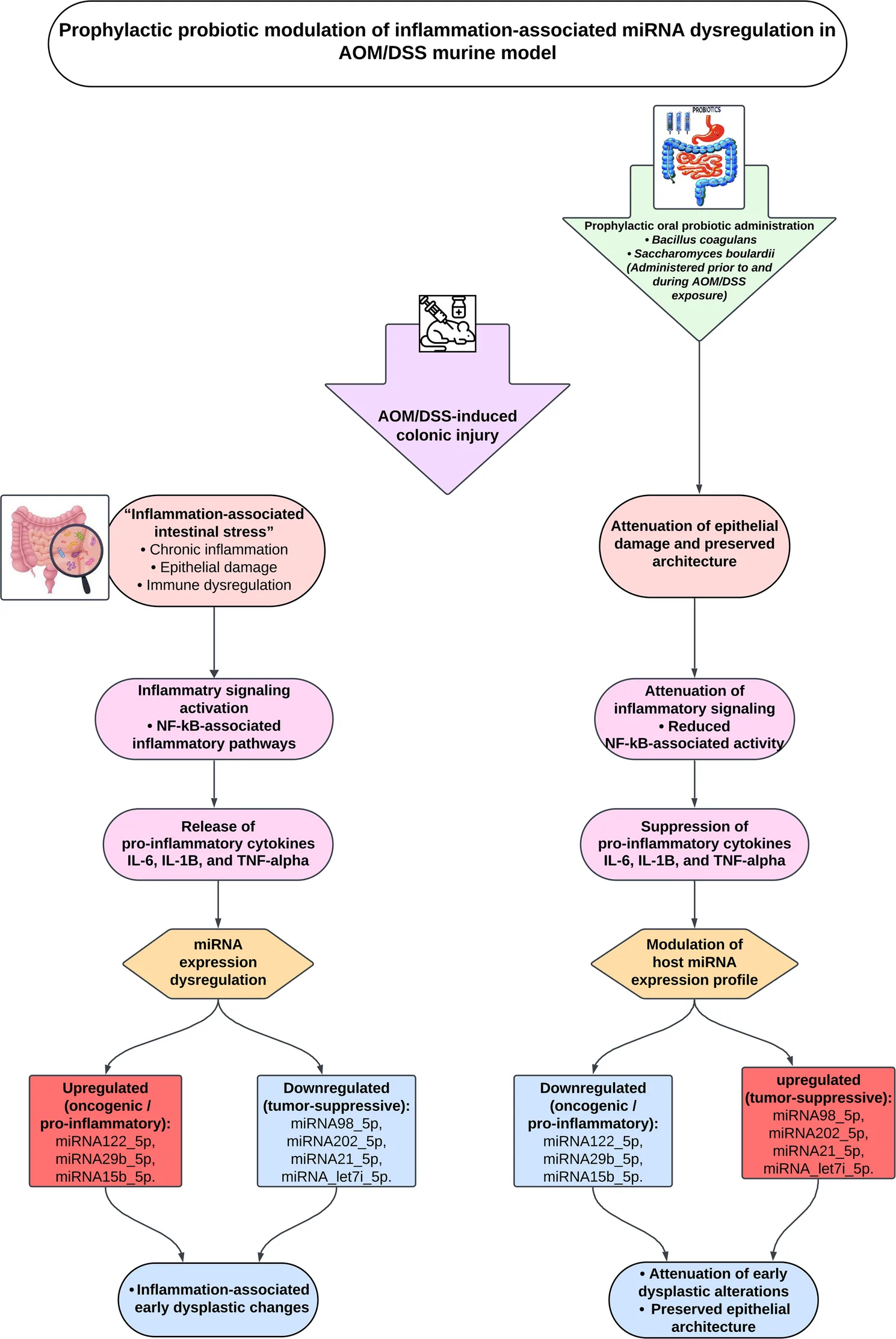

**Supplementary Information:**

The online version contains supplementary material available at https://doi.org/10.1186/s12866-026-05634-z.

## Background

Colorectal cancer (CRC) remains a major global health burden and a leading cause of cancer-related morbidity and mortality worldwide [1, 2]. In Egypt, CRC represents a growing public health concern, emphasizing the importance of preventive strategies targeting early premalignant stages, including colorectal dysplasia [3].

A central driver of colorectal carcinogenesis is chronic inflammation, which promotes epithelial injury, immune dysregulation, and tumor-promoting signaling [4]. In inflammation-associated CRC models, sustained activation of NF-κB signaling promotes a pro-inflammatory microenvironment by increasing the production of cytokines such as IL-6, IL-1β, and TNF-α, thereby facilitating dysplastic transformation [5, 6].

MicroRNAs (miRNAs) are important post-transcriptional regulators of inflammatory signaling, immune responses, epithelial homeostasis, and colorectal carcinogenesis [7, 8]. Several miRNAs, including miR-21, miR-122, and let-7 family members, have been implicated in inflammation-associated carcinogenesis and NF-κB-related signaling [9, 10] supporting the investigation of selected miRNAs as molecular indicators of early inflammation-associated dysplastic changes.

Probiotics have gained attention as potential preventive interventions due to their reported anti-inflammatory, immunomodulatory, and epithelial-protective properties [11, 12]. Although some probiotic effects have been attributed to modulation of the intestinal microbiota, microbiota composition was not assessed in the present study; therefore, such mechanisms are considered only as background evidence. Among probiotic strains, *Weizmannia coagulans* and *Saccharomyces boulardii* have demonstrated properties relevant to intestinal inflammation and epithelial homeostasis [13, 14]. Recent experimental evidence also supports the investigation of prophylactic or pre-administration approaches involving *S. boulardii*-derived interventions in intestinal inflammation. However, the comparative prophylactic effects of *W. coagulans* and *S. boulardii* during early AOM/DSS-induced colorectal dysplasia and their association with host miRNA expression remain insufficiently characterized.

**Fig. 1:**
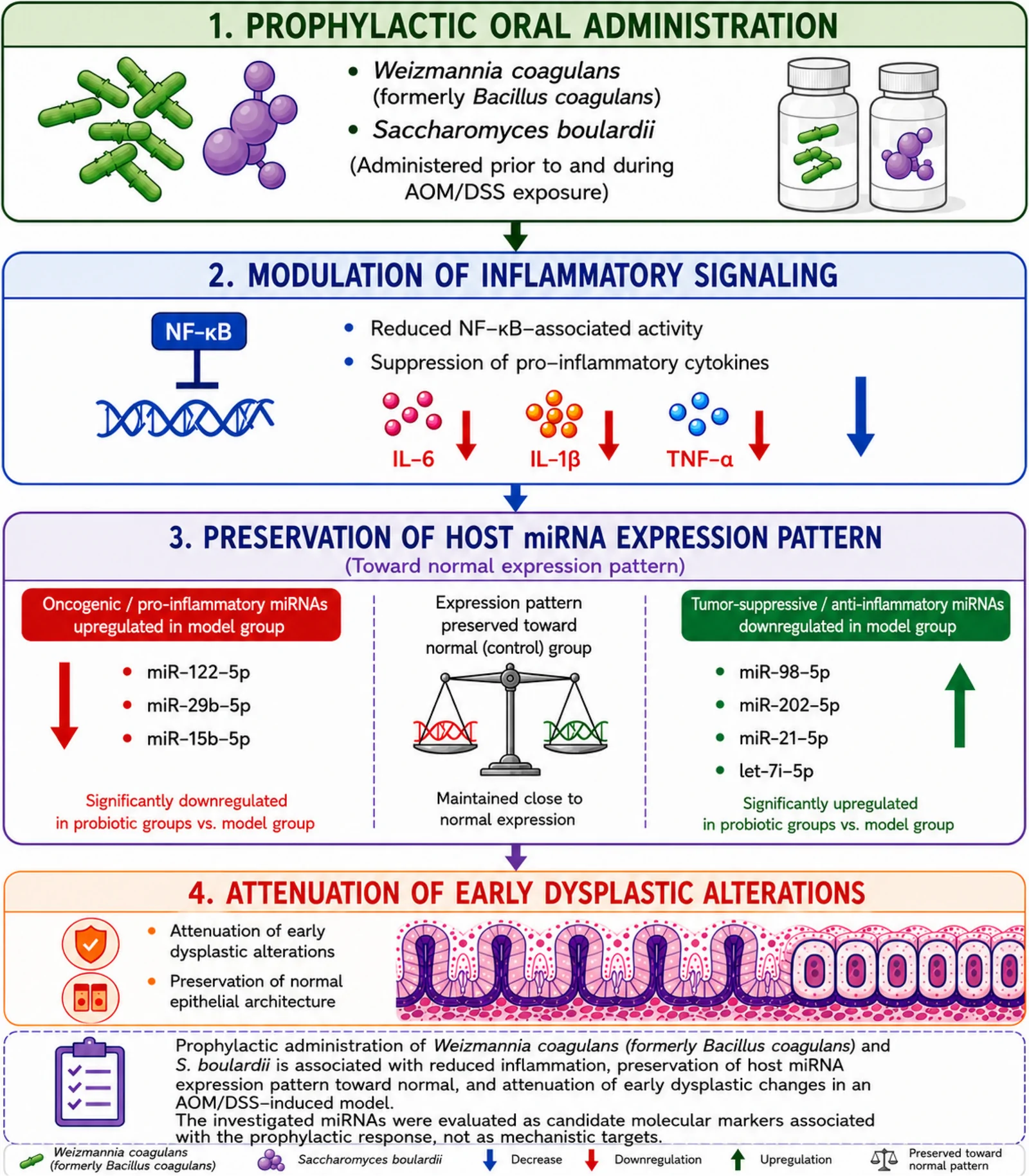
Conceptual overview of the outcomes associated with prophylactic probiotic administration in the AOM/DSS-induced mouse model. The schematic summarizes the observed associations between prophylactic administration of *W. coagulans* and *S. boulardii*, reduced inflammatory responses, preservation of host miRNA expression patterns toward control levels, and attenuation of early dysplastic alterations. The investigated miRNAs are presented as candidate molecular markers associated with the prophylactic response and not as established mechanistic mediators of probiotic action

Accordingly, this study investigated the prophylactic effects of *W. coagulans* and *S. boulardii*, administered before AOM/DSS induction and continued throughout the experiment, on inflammation-associated early colorectal dysplasia. Seven miRNAs (miR-202, let-7i, miR-122, miR-98, miR-29b, miR-21, and miR-15b) were selected through an integrated bioinformatics and literature based strategy based on their differential expression in CRC across multiple public databases, their relevance to immune regulatory mechanisms, and pathway enrichment analyses implicating them in cancer-related and immunomodulatory pathways, together with predicted interactions with inflammation-related molecular targets [15–19]. The study evaluated whether probiotic prophylaxis was associated with attenuation of inflammatory and early dysplastic changes and preservation of miRNA expression patterns. The selected miRNAs were investigated as candidate molecular markers associated with the prophylactic response rather than as established mechanistic targets of probiotic action, and neither miRNA regulation nor microbiota modulation was assumed to represent a direct mechanism underlying the observed effects (Fig. 1).

## Materials and methods

### Bioinformatics analysis and miRNA selection

#### Retrieval of CRC-linked miRNA panel

To investigate the role of miRNAs in colorectal carcinogenesis, a bioinformatics workflow was applied to identify miRNAs associated with CRC-related inflammation, epithelial damage, and immune regulation. Initial screening was performed using the OncomiR database, (version 2.0,, https://www.oncomir.umn.edu/omcd/basic_search.php accessed November 2025), to identify miRNAs differentially expressed in CRC tissues compared with controls with the following parameters: Cancer type: Colorectal adenocarcinoma, Tissue comparison: Tumor vs. Normal, Threshold: miRNAs with |log₂ fold‑change| > 1.0 and adjusted p‑value < 0.05 in at least two independent datasets were retained. Seven miRNAs—hsa-miR-122, hsa-miR-29b, hsa-miR-15b, hsa-miR-202, hsa-miR-98, hsa-miR-21, and hsa-miR-let7i—were selected based on: (a) Consistent differential expression across ≥ 2 independent CRC datasets in OncomiR; (b) Literature‑reported involvement in inflammation‑driven pathways (NF‑κB, TGF‑β, p53). (c) Relevance to colitis‑associated carcinogenesis based on prior experimental evidence.

Expression dynamics of the selected miRNAs across CRC stages were further examined using the Tissue Atlas databases version 2.5 (https://ccb-web.cs.uni-saarland.de/tissueatlas2, accessed 25th December 2025) and dbDEMC databases, version 3 (https://www.biosino.org/dbDEMC/index, accessed December 2025) (Additional Figures S1–S4). Parameters included: (1) Stage‑wise comparison (normal, adenoma, carcinoma), (2) Threshold: FDR‑adjusted *p* < 0.05 for stage‑specific differential expression.

Associations between these miRNAs and colonic inflammation or dysplasia were evaluated using the Immune-miR database, version 1.2 (http://www.biominingbu.org/immunemir/, accessed December 2025) with default parameters (*p* < 0.05, enrichment score > 2.0). (Additional Figure S5).

Pathway enrichment analysis was conducted using DIANA-miRPath v3.0 (https://dianalab.e-ce.uth.gr/html/mirpathv3/ ,accessed January 2026) to map the selected miRNAs to cancer- and inflammation-related pathways, including NF-κB, TGF-β, p53, FOXO, Hippo, and AMPK signaling pathways (Additional Figure S6) with the following settings: Organism: Homo sapiens, Pathways: KEGG, Statistics: microT threshold = 0.8, p‑value threshold = 0.05, FDR correction applied. Enriched pathways included NF‑κB, TGF‑β, p53, FOXO, Hippo, and AMPK signaling.

Based on validated miRNA–target interactions, IL-6, IL-1β, and TNF-α were identified as key downstream effector proteins for experimental validation (Additional Figures S7–S9). These mediators have been widely reported to participate in immune and inflammatory signaling relevant to CRC progression, as demonstrated in previous studies [20–23].

All in all, The miRNAs were chosen based on a combination of literature review and bioinformatics predictions. We conducted a thorough review of existing literature to identify miRNAs that have been implicated in colorectal carcinogenesis and inflammation. This included a focus on miRNAs known to respond to microbial and probiotic signals, such as miR-122, miR-21, and members of the Let-7 family. Database access dates, selection criteria, and enrichment parameters are detailed in Additional File 1 and Additional Table S1.

#### Experimental animals

Thirty-two male BALB/c mice (6–8 weeks old; body weight 22 ± 2 g) were obtained from the animal house of Nile Pharmaceuticals Company (Cairo, Egypt), a licensed commercial breeder. Animals were housed under standard laboratory conditions (22 ± 2 °C, 12 h light/dark cycle) with ad libitum access to standard chow and water. All experimental procedures were conducted in accordance with institutional and national guidelines for the care and use of laboratory animals. The study protocol was reviewed and approved by the Research Ethics Committee (REC), Faculty of Medicine, Ain Shams University (Protocol No. FMASU R218/2022; approval date: 24 November 2022; Federal Wide Assurance No. FWA 000017585). Informed consent was not applicable, as the study was performed on laboratory mice and did not involve client-owned animals.

#### Experimental design and grouping

Mice were randomly assigned into four experimental groups (*n* = 8 per group):

1. Control group (*n* = 8): maintained on a normal chow diet and received intraperitoneal (IP) injection of saline without additional treatment.
2. AOM/DSS model group (*n* = 8): subjected to azoxymethane (AOM) and dextran sulfate sodium (DSS) to induce colitis-associated dysplasia.
3. Prophylactic *Saccharomyces boulardii* group: received *S. boulardii* (*n* = 8); each mouse was administered 150 mg/kg dissolved in water by oral gavage, starting one week prior to AOM injection and continuing throughout the experiment alongside 2% DSS.
4. Prophylactic *Weizmannia coagulans* group (*n* = 8): received *W. coagulans*; each mouse was administered 1 × 10⁹ spores dissolved in water by oral gavage daily, starting one week prior to AOM injection and continuing throughout the experiment alongside 2% DSS.

Within each group, four mice were sacrificed at week 12 and four at week 16, representing early inflammatory/dysplastic and more advanced dysplastic stages, respectively. This design allowed evaluation of probiotic effects across early and established stages of AOM/DSS-induced dysplasia (Additional *Figure. 10*). Probiotic-only, AOM-only, and DSS-only control groups were not included to minimize animal use and because the study focused on prophylactic effects rather than baseline probiotic physiology; this is acknowledged as a limitation. In addition, the absence of gut microbiota profiling limits the mechanistic interpretation of the observed probiotic-associated inflammatory and miRNA changes.

#### Induction of colitis-associated dysplasia

Colorectal dysplasia was induced using an established azoxymethane/dextran sulfate sodium (AOM/DSS) protocol. Mice received a single intraperitoneal injection of azoxymethane (AOM; 10 mg/kg body weight; Sigma-Aldrich, Germany) dissolved in sterile 0.9% saline. One week later, dextran sulfate sodium (DSS; 2%, molecular weight 36–50 kDa; Sigma-Aldrich, Germany) was administered in drinking water for seven consecutive days, after which animals were returned to normal water [24, 25].

Animals were sacrificed at weeks 12 and 16 to assess different stages of inflammation-associated colorectal dysplasia. Previous studies have shown that preneoplastic lesions, including aberrant crypt foci and epithelial hyperplasia/dysplasia, are detectable by week 12, whereas more established dysplastic changes emerge at later time points in the AOM/DSS model [26]. Accordingly, week 12 was used to confirm early pathological changes, while week 16 was selected to evaluate sustained and more pronounced effects of prophylactic probiotic intervention on established dysplasia.

#### Probiotic strains, culture conditions, and administration

The probiotic strains *Weizmannia coagulans* ATCC 7050 (Prescribed For Life^®^, Fredericksburg, TX, USA) and *Saccharomyces boulardii* ATCC MYA-796 (FloraMax^®^, Richmond, VA, USA) were cultured under strain-specific conditions. *W. coagulans* was grown in nutrient broth, while *S. boulardii* was cultured in a yeast-supportive medium, following optimized protocols to maintain viability during gastrointestinal transit. Viability and cell counts were confirmed prior to administration using the standard plate-counting method, calculated as:

CFU/mL = number of colonies × dilution factor [27].

Final concentrations were adjusted to 5 × 10⁹ CFU/mL for *W. coagulans* and 8 × 10⁷ CFU/mL for *S. boulardii*. Each mouse received 200 µL of freshly prepared probiotic suspension by oral gavage once daily, corresponding to a dose of 1 × 10⁹ CFU/mouse/day for *W. coagulans* and 1 × 10⁷ CFU/mouse/day for *S. boulardii*. Based on the mean baseline body weight of 22 g, these doses correspond to approximately 4.55 × 10¹⁰ CFU/kg/day for *W. coagulans* and 4.55 × 10⁸ CFU/kg/day for *S. boulardii*. Probiotic administration began one week before azoxymethane (AOM) injection and continued throughout dextran sulfate sodium (DSS) exposure. Preparations were stored at 4 °C for less than 24 h before use. Although probiotic viability was confirmed before administration via plate counting, we did not perform post‑administration fecal or tissue cultures to verify intestinal colonization or engraftment. Therefore, the effects reported herein are based on daily probiotic delivery rather than confirmed persistent colonization.

#### Sample collection and tissue processing

Mice were anesthetized with sodium pentobarbital (45 mg/kg, intraperitoneal) and euthanized by cervical dislocation. Blood samples were obtained from the retro-orbital veins and centrifuged at 3000 rpm for 10 min to separate the serum. Serum samples were collected and stored at − 80 °C for subsequent biochemical analysis.

Following sacrifice, a laparotomy was performed and the colon was carefully dissected. The colon was identified from the ileocecal junction to approximately 1.5 cm above the anal orifice, and its length was measured in centimeters. The colon was then divided into three equal segments: proximal, middle, and distal. From each mouse, the distal colon was divided into two portions: one half was sectioned into small transverse segments (~ 0.5 cm) and processed for histopathological and immunohistochemical analyses, while the remaining half was stored at − 80 °C for subsequent molecular assays.

All experimental procedures, including biochemical assays, histopathological evaluation, immunohistochemistry, and molecular analyses (miRNA extraction and RT-qPCR), were performed on samples obtained from all 32 mice at both sacrifice time points (weeks 12 and 16) (Additional *Table* S2, Additional *Figure. 11*). For clarity and consistency, quantitative data are presented for the 16-week time point only, as this stage represents well-established inflammation-associated dysplastic alterations and allows a more robust evaluation of probiotic-mediated attenuation. Data from week 12 were used to confirm early pathological trends but were not included in the final quantitative presentation (Experimental design and sample allocation detail in Additional table S2).

#### Biochemical analysis of serum samples

Blood samples were collected from all 32 mice at sacrifice and processed to obtain serum. Serum samples were analyzed for a range of biochemical parameters, including protein markers (albumin and total protein), inflammatory markers (C-reactive protein [CRP], IL-1β, and IL-6), and tumor-associated markers (CA19-9, carcinoembryonic antigen [CEA], and alpha-fetoprotein). In addition, liver and kidney function tests were performed, alongside metabolic assessments of serum glucose levels and lipid profiles. All biochemical analyses were conducted on an individual sample basis.

Serum levels of IL-6 and IL-1β were quantified using commercially available ELISA kits (Mouse IL-6 ELISA Kit, Cat. No. E0079m; Mouse IL-1β ELISA Kit, Cat. No. E0563m; EIAAB Science Inc., China) according to the manufacturer’s instructions. Briefly, 100 µL of serum or standard was added to antibody-coated wells and incubated for 90 min at 37 °C. After washing four times with wash buffer, 100 µL of biotinylated detection antibody was added and incubated for 60 min at 37 °C. Wells were washed again, followed by addition of 100 µL of streptavidin-HRP conjugate and incubation for 30 min at 37 °C. After a final wash, 90 µL of TMB substrate was added and incubated for 15 min at 37 °C in the dark. The reaction was stopped with 50 µL of stop solution, and absorbance was measured at 450 nm using a microplate reader. A standard curve was generated using seven serial dilutions of recombinant cytokine standards provided in the kit. Absorbance values of standards were plotted against their known concentrations, and a four-parameter logistic (4PL) regression model was applied to fit the curve. The concentration of cytokines in each sample was interpolated from the standard curve using the measured absorbance. All assays were performed in duplicate, and results were normalized to total protein content determined by the Bradford method.

### Histopathological examination

#### Tissue collection and processing

For histopathological and immunohistochemical analyses, 4 mice per group per time point (total *n* = 32 across all experimental groups) were examined at the selected time point. From each mouse, paraffin sections were prepared four representative slides per group were evaluated. Quantitative assessment was performed by analyzing five non-overlapping microscopic fields per slide, yielding a total of 20 fields per group to minimize sampling bias. Representative photomicrographs reflect consistent findings observed across all examined sections. Distal colon tissues were fixed in 10% neutral-buffered formalin for five days, embedded in paraffin, sectioned at 5 μm, and stained with H&E, Masson’s trichrome, and Alcian blue–PAS. Histological specimens were prepared, examined, and photographed at the Histology Department Faculty of Medicine ASU, using a Zeiss Primo Star microscope and a Canon *EOS* 1100D *Digital* SLR camera.

#### Histological staining

Paraffin sections were stained using the following methods:

- Hematoxylin and Eosin (H&E): for general assessment of tissue architecture and cellular morphology.
- Masson’s Trichrome: to evaluate collagen deposition and fibrotic changes.
- Alcian Blue–Periodic Acid–Schiff (AB-PAS): to identify goblet cells and mucopolysaccharide content [28].

#### Histopathological scoring of H&E-stained sections

Examination of H&E-stained distal colon sections was performed to score histopathological changes at 16 weeks. The evaluated parameters included dilated crypts, obliterated crypts, areas of ill-defined basement membrane, invasion of colonic epithelium into the lamina propria, basal epithelial hyperplasia, dysplasia, increased nuclear-to-cytoplasmic ratio, irregular nuclear outlines, inflammatory cell infiltration in the submucosa, loss of colonic architecture, and epithelial sloughing. Scoring was performed independently by two blinded evaluators in five randomly selected non-overlapping fields per mouse, resulting in 20 fields per experimental group. The frequency distribution (%) of each histopathological parameter was calculated, and statistical analysis was performed using the Chi-square test. Pairwise comparisons were corrected for multiple testing using the Bonferroni method.

#### Dysplasia scoring and histomorphometric analysis

In addition to qualitative histopathological scoring, quantitative histomorphometric analysis was performed to objectively assess structural and inflammatory changes in the distal colon. Colonic dysplasia was evaluated using a semi-quantitative scoring system ranging from 0 to 3 (0 = no dysplasia, 1 = mild, 2 = moderate, 3 = severe). Scoring was performed independently by two experienced histopathologists blinded to experimental group allocation. Inter-observer agreement was assessed, yielding a kappa coefficient of 0.84. Images were captured using a Leica DM microscope and analyzed using Leica LAS X Core software. All images were acquired using identical exposure and illumination settings. Positive DAB staining was quantified by applying a fixed intensity threshold, and the percentage of positively stained area was calculated within predefined regions of interest. Parameters assessed included the number of goblet cells per high-power field to evaluate mucosal secretory activity, the area percentage of collagen fibers to measure fibrotic changes, and the area percentage of TNF-α expression to determine the extent of inflammatory responses. For each group, five different fields from four different sections were analyzed to ensure statistical reliability and minimize sampling bias.

#### Immunohistochemistry for TNF-α

Immunohistochemistry for TNF-α expression was conducted on paraffin sections mounted on positively charged slides, enhancing tissue adhesion during staining. Avidin-biotin peroxidase staining was applied to detect inflammatory cells expressing TNF-α. Sections were deparaffinized in xylene and hydrated in descending concentrations of alcohol. Then were incubated in 0.5% hydrogen peroxide for 10 min (to block endogenous peroxidase) and washed in phosphate buffer saline (PBS) (2 times, 10 min each). Antigen unmasking was done using citrated buffer (pH 6) for 15 min, then sections were rinsed in PBS, incubated in 0.1% bovine serum albumin in PBS. Washing in PBS was followed by incubation in the primary antibody. The antibodies against TNF-α (Abcam, Cat. No. TNFA/1172, BSA and Azide-free, ab269772) were used at a dilution of 1:200.

The slides were incubated overnight at 4 °C in a humidified chamber. After three cycles of 5 min wash in PBS, sections were then incubated with horseradish peroxidase-conjugated secondary antibody for one hour at room temperature. Then, sections were washed with PBS, counterstained with hematoxylin (to visualize cell nuclei), dehydrated in ascending concentrations of alcohol, cleared in xylene, and mounted with DPX. Positive control consisted of tissue sections from the mouse stomach, while negative control was prepared by replacing the primary antibody with PBS to confirm specificity [29].

TNF-α immunoreactivity was evaluated by two blinded observers using a semi-quantitative scoring system based on the intensity and extent of staining. Staining intensity was scored as: 0 (negative), 1 (weak), 2 (moderate), or 3 (strong). The percentage of positive cells was assessed as: 0 (0%), 1 (1–25%), 2 (26–50%), 3 (51–75%), or 4 (76–100%). A combined immunoreactivity score (IRS) was calculated by multiplying the intensity score by the percentage score, resulting in a range of 0–12. For digital quantification, five non-overlapping high-power fields (×400) per section were captured using a Leica DM2500 microscope and analyzed with Leica LAS X software. A fixed intensity threshold was applied to quantify the DAB-positive area, expressed as the percentage of positively stained area per field.

#### miRNA extraction and quantitative RT-qPCR analysis

Total RNA, including small RNA fractions, was extracted from distal colon tissue samples using the miRNeasy Mini Kit (Qiagen, Cat. No. 217004) according to the manufacturer’s instructions. RNA concentration and purity were assessed using a NanoDrop spectrophotometer (Thermo Scientific, USA), and samples with A260/A280 ratios between 1.8 and 2.0 were considered suitable for downstream analysis. Reverse transcription was performed immediately after RNA extraction using the miScript II RT Kit (Qiagen, Cat. Nos. 218160 and 218161), which is optimized for miRNA cDNA synthesis. Reverse transcription reactions were carried out in a Thermo Hybaid PCR Express Thermal Cycler (Thermo Fisher Scientific, USA) following the manufacturer’s protocol.

Quantitative miRNA expression analysis was conducted using the miRCURY SYBR^®^ Green PCR Kit (Qiagen, Cat. No. 339345) in combination with the miRCURY LNA™ miRNA Custom PCR Panel (Qiagen, Cat. Nos. 339330 and 339332). The panel contains manufacturer-validated LNA-enhanced miRNA-specific primer assays for the targeted miRNAs. Reactions were performed on an Applied Biosystems 7500 Real-Time PCR System (Applied Biosystems, USA) using SYBR Green chemistry. Cel-miR-39 was added as an external spike-in control during RNA extraction to monitor extraction efficiency. U6 small nuclear RNA (snRNA) served as the endogenous reference gene for normalization of miRNA expression levels. Each qPCR reaction was performed in duplicate to ensure technical reproducibility. All primer sequences and assay configurations are proprietary to Qiagen and are optimized for high specificity and PCR efficiency (typically > 90% as reported by the manufacturer).

Thermal cycling conditions consisted of an initial denaturation at 95 °C for 2 min, followed by 40 cycles of denaturation at 95 °C for 5 s and annealing/extension at 56 °C for 30 s. Threshold cycle (Ct) values were determined using Applied Biosystems 7500 Software v2.3, and Ct values > 36 were considered negative. Melting-curve analysis was performed at the end of each run to verify amplification specificity. PCR amplification efficiency was not independently calculated in this study; therefore, manufacturer-validated LNA primer assays were used, and assay specificity was ensured by melting-curve analysis. Relative miRNA expression levels were calculated using the 2⁻ΔΔCt (Livak) method [30] and expressed as fold change relative to the control group.

### Statistical analysis

The sample size was determined based on ethical reduction principles, prior pilot data, and power considerations to ensure adequate detection of probiotic effects on primary inflammatory and molecular outcomes. All statistical analyses were performed using SPSS software version 25.0 (IBM Corp., Chicago, IL, USA). Data are expressed as mean ± standard deviation (SD) with corresponding 95% confidence intervals (CI). Data normality was assessed using the Shapiro–Wilk test. For normally distributed variables, group comparisons were performed using one-way ANOVA. When ANOVA indicated significant variance, Tukey’s honestly significant difference (HSD) post‑hoc test was applied for pairwise comparisons. For analyses involving multiple hypothesis testing, Bonferroni correction was applied where appropriate to control for type I error. For non‑normally distributed data or ordinal variables (e.g., histopathological scores), the Kruskal–Wallis test followed by Dunn’s post‑hoc test with Bonferroni adjustment was used. Correlation analyses were performed using Spearman’s rank correlation coefficient due to non-normal distribution of correlation variables. Statistical significance was set at *p* < 0.05. For correlation analyses involving multiple comparisons (e.g., 7 miRNAs × 15 biochemical parameters), p‑values were adjusted using the Benjamini–Hochberg false discovery rate (FDR) procedure. Adjusted p‑values (q‑values) are reported in Additional tables, and significance was defined as q < 0.05.

## Results

### Body weight changes in the AOM/DSS-induced mouse model

Baseline body weights did not differ significantly among the experimental groups (*p* > 0.05). As shown in Additional Fig. 12, mice in the AOM/DSS model group exhibited a significant reduction in body weight at both week 12 and week 16 compared with the control group (*p* < 0.05), reflecting disease-associated systemic deterioration. In contrast, mice receiving prophylactic *Saccharomyces boulardii* or *Weizmannia coagulans* did not show significant differences in body weight compared with controls at either time point (*p* > 0.05). Both probiotic-treated groups maintained significantly higher body weights at weeks 12 and 16 when compared with the AOM/DSS model group (*p* < 0.05), indicating attenuation of AOM/DSS-induced weight loss. No statistically significant differences in body weight were observed between the two probiotic prophylactic groups at either week 12 or week 16 (*p* > 0.05).

### Biochemical parameters

**Fig. 2:**
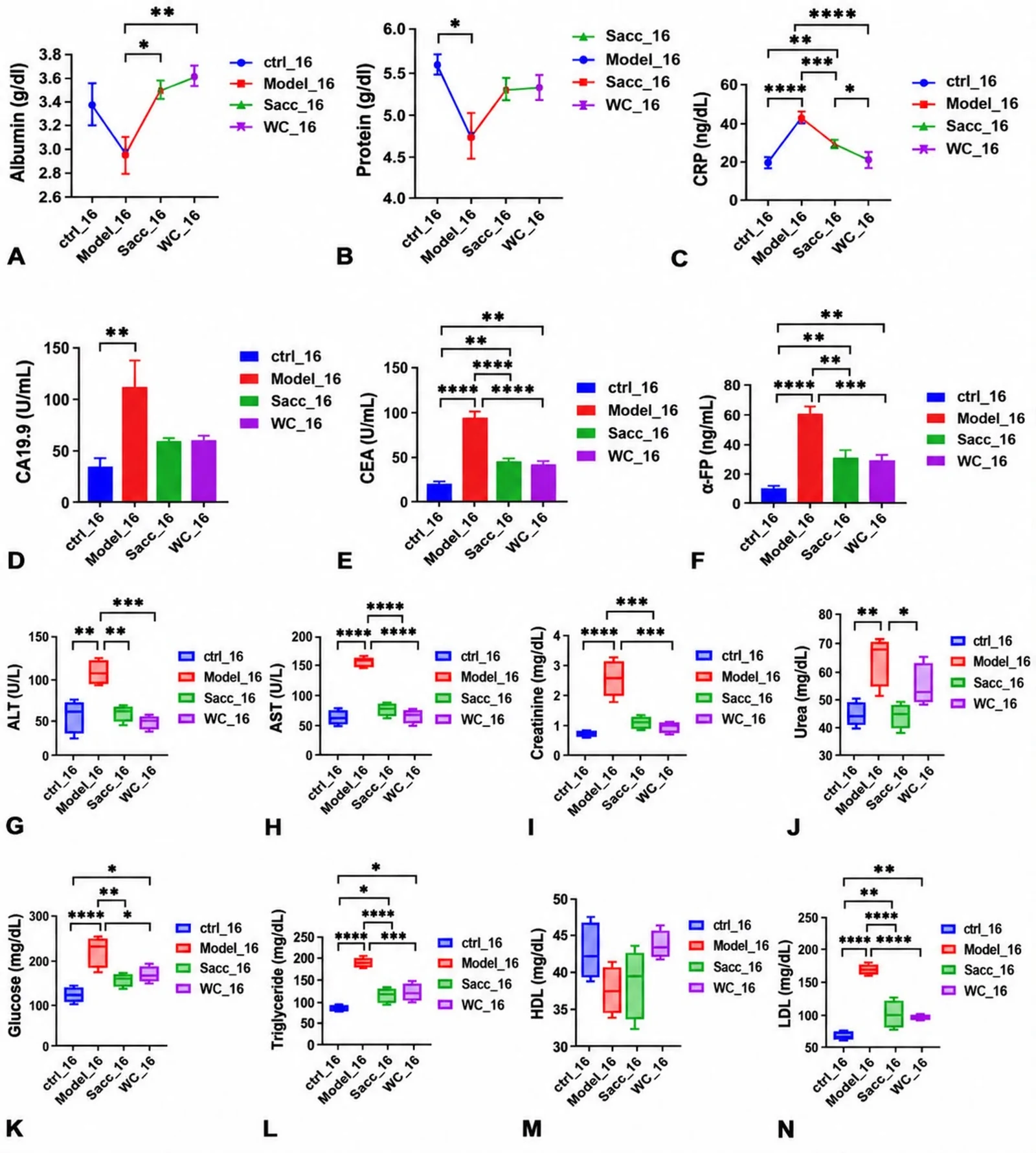
Impact of AOM/DSS-induced dysplastic changes and prophylactic probiotic administration on serum biochemical parameters at 16 weeks. Serum biochemical profiles are shown for control (ctrl_16), AOM/DSS model (Model_16), *S. boulardii* prophylactic (Sacc_16), and *W. coagulans* prophylactic (WC_16) groups. Parameters include albumin (**A**), total protein (**B**), C-reactive protein (CRP; **C**), CA19.9 (**D**), carcinoembryonic antigen (CEA; **E**), alpha-fetoprotein (AFP; **F**), alanine aminotransferase (ALT; **G**), aspartate aminotransferase (AST; **H**), creatinine (**I**), urea (**J**), glucose (**K**), triglycerides (**L**), high-density lipoprotein (HDL; **M**), and low-density lipoprotein (LDL; **N**). Data are presented as mean ± SD (*n* = 4 mice per group). Statistical analysis was performed using one-way ANOVA followed by Tukey’s multiple comparison test. Significance levels are indicated as *p < 0.05, **p < 0.01, ***p < 0.001, ****p < 0.0001

At 16 weeks, serum biochemical assessment demonstrated significant alterations in inflammatory, tumor-associated, organ-function, and metabolic parameters following AOM/DSS exposure. Significant overall group differences were observed for albumin, total protein, CRP, CA19.9, CEA, AFP, IL-1β, IL-6, ALT, AST, creatinine, urea, glucose, triglycerides, and LDL cholesterol (all *p* < 0.01), whereas HDL levels did not differ significantly among groups (*p* = 0.07) (Fig. 2; Table 1; Additional Table S3).

Regarding the inflammatory and tumor-associated profile, the AOM/DSS model group exhibited significantly elevated CRP, IL-1β, IL-6, CA19.9, CEA, and AFP levels compared with controls (all *p* < 0.01). Notably, IL-1β increased from 25.00 ± 17.98 ng/L in controls to 93.75 ± 14.17 ng/L in the AOM/DSS model group, while IL-6 increased from 143.3 ± 35.02 to 365.0 ± 36.29 pg/mg protein, respectively. Prophylactic administration of *S. boulardii* or *W. coagulans* markedly attenuated these inflammatory alterations, with IL-1β levels of 40.75 ± 6.55 and 42.75 ± 6.29 ng/L, respectively, and IL-6 levels of 159.3 ± 9.87 and 147.3 ± 9.28 pg/mg protein, respectively; these values were not significantly different from controls. CEA and AFP, however, remained mildly elevated in both probiotic groups (*p* < 0.05). CRP remained significantly higher in the *S. boulardii* group than in controls (*p* < 0.01), while direct comparison of the probiotic groups demonstrated significantly lower CRP levels in the *W. coagulans* group (*p* < 0.05) (Fig. 2C–F; Table 1).

For protein and organ-function parameters, AOM/DSS exposure was associated with significantly elevated ALT, AST, creatinine, and urea levels compared with controls (all *p* < 0.01), accompanied by a modest reduction in total protein (*p* = 0.02) and a non-significant decreasing trend in albumin (*p* = 0.07). Both probiotic groups exhibited profiles largely comparable to controls for albumin, total protein, ALT, AST, creatinine, and urea. Compared with the model group, both probiotic interventions were associated with significant improvements in albumin, ALT, AST, and creatinine (*p* < 0.05), whereas a significant reduction in urea was observed only in the *S. boulardii* group (Fig. 2A, B, G–J; Additional Table S3).

Regarding the metabolic profile, glucose, triglycerides, and LDL cholesterol were significantly elevated in the AOM/DSS model group compared with controls (all *p* < 0.01), whereas HDL levels did not differ significantly among groups (*p* = 0.07). Both probiotic groups showed significant improvements in glucose and triglyceride levels relative to the model group (*p* < 0.05), although triglycerides and LDL cholesterol remained mildly elevated compared with controls. Glucose levels also remained significantly different between the *W. coagulans* and control groups (*p* < 0.05) (Fig. 2K–N; Additional Table S3).

**Fig. 3:**
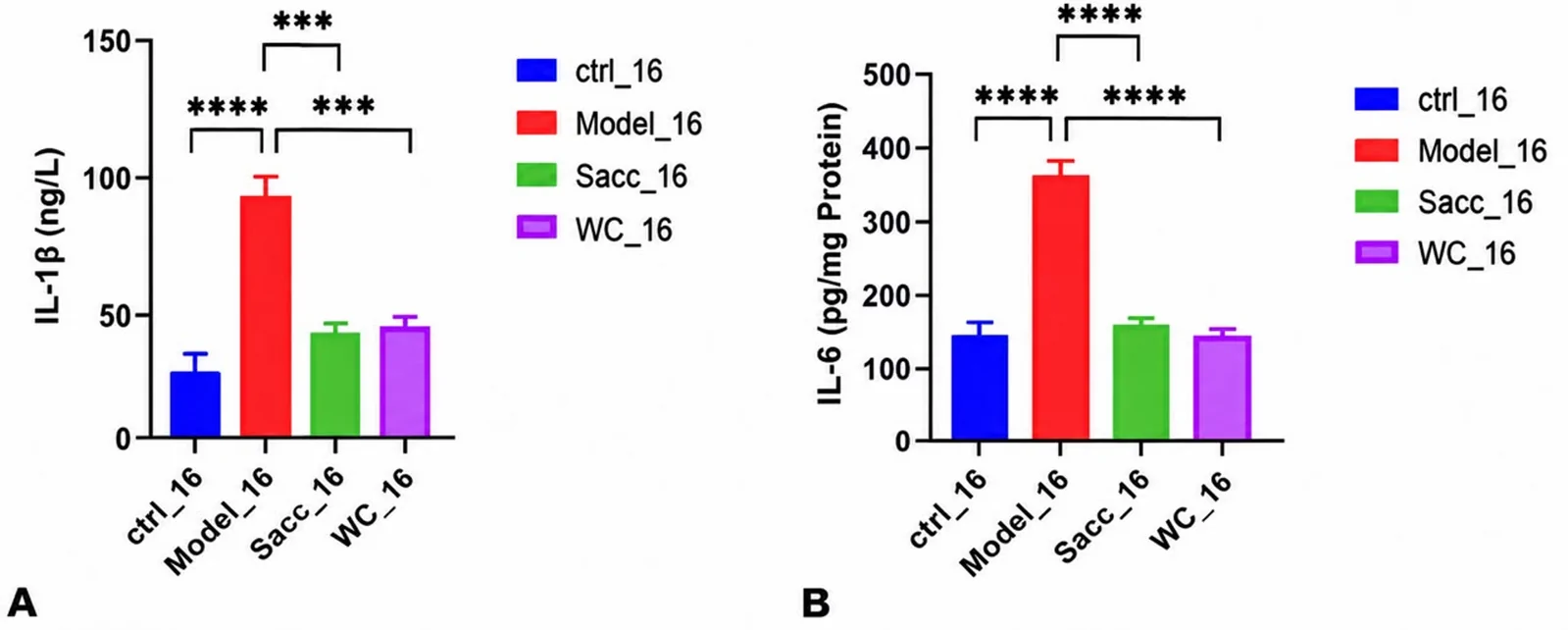
Pro-inflammatory cytokine levels at 16 weeks. Serum IL-1β (**A**) and IL-6 (**B**) levels in control (ctrl_16), AOM/DSS model (Model_16), S. boulardii prophylactic (Sacc_16), and W. coagulans prophylactic (WC_16) groups. Data are mean ± SD (n = 4/group). One-way ANOVA with Tukey’s post hoc test; *p < 0.05, **p < 0.01, ***p < 0.001

Overall, prophylactic administration of *S. boulardii* and *W. coagulans* was associated with attenuation of AOM/DSS-related inflammatory, tumor-associated, organ-function, and metabolic alterations, with broadly comparable biochemical profiles between the two probiotic groups (Fig. 2; Table 1; Additional Table S3)(Fig. 3).

**Table 1:** Serum IL-1β and IL-6 levels at 16 weeks across experimental groups

Groups	Control gp 16 wks	AOM/DSS-Induced Model gp 16 wks	*S. boulardii* Prophylactic gp 16 wks	*W. coagulans* Prophylactic gp 16 wks	F	*P*-Value
Pro-inflammatory cytokine	mean ± SD 95% CI	mean ± SD 95% CI	mean ± SD 95% CI	mean ± SD 95% CI		
IL-1β (ng/L)	25.00 ± 17.98 (-3.61-53.61)	93.75 ± 14.17 (71.19–116.30)	40.75 ± 6.55 (30.32–51.17)	42.75 ± 6.29 (32.74–52.76)	23.52	0.000*^a^
						0.000*^b^
						0.316^c^
						0.228^d^
						0.000*^e^
						0.000*^f^
						0.995^g^
IL-6 (pg/mg Protein)	143.3 ± 35.02 (87.53-198.97)	365.0 ± 36.29 (307.26-422.74)	159.3 ± 9.87 (143.53-174.97)	147.3 ± 9.28 (132.47-162.03)	68.13	0.000*^a^
						0.000*^b^
						0.822^c^
						0.996^d^
						0.000*^e^
						0.000*^f^
						0.914^g^

Serum concentrations of IL-1β (ng/L) and IL-6 (pg/mg protein) measured at 16 weeks in control, AOM/DSS-induced model, *Saccharomyces boulardii* prophylactic, and *Weizmannia coagulans* prophylactic groups (n = 4 mice per group). Data are presented as mean ± SD with 95% confidence intervals. Statistical analysis was performed using one-way ANOVA; Tukey’s post-hoc comparisons included overall group comparison (a), control vs model (b), control vs *S. boulardii* (c), control vs *W. coagulans* (d), model vs *S. boulardii* (e), model vs *W. coagulans* (f), and *S. boulardii* vs *W. coagulans* (g)

*A* p* value < 0.05 was considered statistically significant

### Histopathological results

**Fig. 4:**
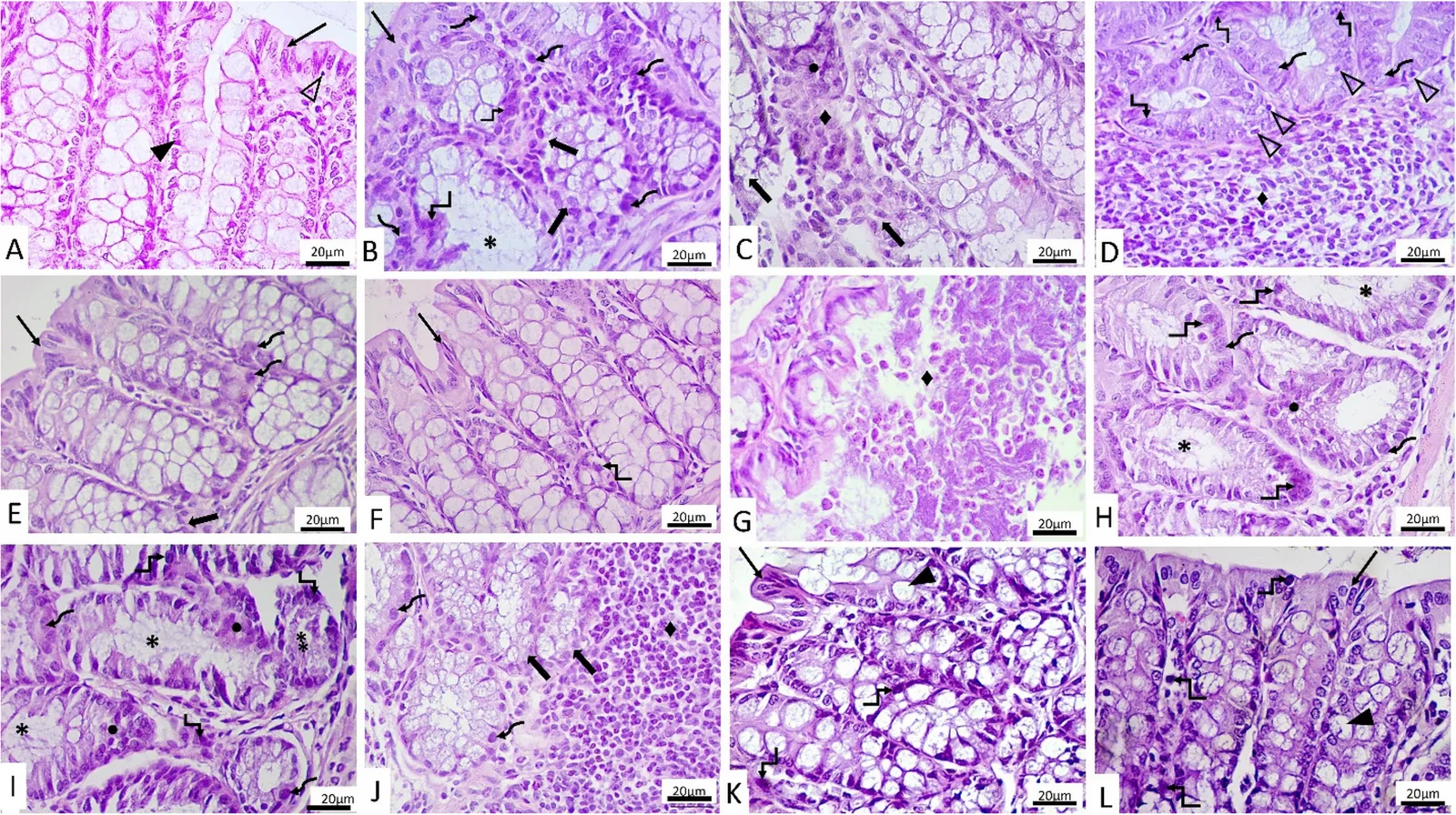
Representative Photomicrographs of the Distal Colon Across Different Groups; **A **Group I (Control), **B**-**D** Model Group at 12 Weeks, **E** S. boulardii- Prophylactic Group at 12 Weeks, **F** W. coagulans- Prophylactic Group at 12 Weeks, **G**-**J** Model Group at 16 Weeks, **K** S. boulardii- Prophylactic Group at 16 Weeks, **L** W. coagulans- Prophylactic Group at 16 Weeks. Key features include columnar absorptive cells (↑) with acidophilic cytoplasm and basal oval nuclei; goblet cells (▲) with vacuolated cytoplasm and basal nuclei; intraepithelial lymphocytes (∆); ill-defined basement membrane (thick arrow); expansion of crypt lumen (*); nuclear dysplasia with an increased nuclear-to-cytoplasmic ratio (curved arrow); irregular nuclear outlines (elbow arrow); infiltration of colonic epithelial cells and inflammatory cells into the lamina propria (♦); crowded cells and basal colonic epithelial hyperplasia (●); and obliterated crypt lumens (**). [H&E staining x400, scale bar: 20 µm]

Histological evaluation of distal colon sections demonstrated progressive alterations in mucosal architecture and dysplastic features following AOM/DSS exposure. At 12 weeks, control sections showed preserved mucosal architecture with intact crypts and abundant goblet cells (Fig. 4A). In contrast, the AOM/DSS model group exhibited crypt dilation, goblet cell depletion, ill-defined basement membranes, epithelial invasion into the lamina propria, basal epithelial hyperplasia, dysplastic changes characterized by increased nuclear-to-cytoplasmic ratio and irregular nuclear outlines, and prominent inflammatory cell infiltration (Fig. 4B–D). The *S. boulardii* prophylactic group showed residual pathological features, including ill-defined basement membranes and epithelial dysplasia (Fig. 4E), whereas the *W. coagulans* group showed occasional irregular nuclear outlines with reduced severity compared with the model group (Fig. 4F). By 16 weeks, the AOM/DSS model group displayed more pronounced disruption of colonic architecture, including epithelial sloughing, crypt hyperplasia, cystic dilation or obliteration, persistent dysplastic changes, poorly defined basement membranes, and extensive inflammatory cell infiltration (Fig. 4G–J). In comparison, both prophylactic probiotic groups showed better preservation of colonic architecture and less severe dysplastic alterations, although occasional irregular nuclear outlines persisted (Fig. 4K, L). Quantitative histopathological assessment at 16 weeks supported these observations, demonstrating significant group differences in several pathological features, including obliterated crypts, ill-defined basement membranes, epithelial invasion, basal epithelial hyperplasia, dysplasia, increased nuclear-to-cytoplasmic ratio, irregular nuclear outlines, inflammatory cell infiltration, loss of colonic architecture, and epithelial sloughing (Table 2).

**Fig. 5:**
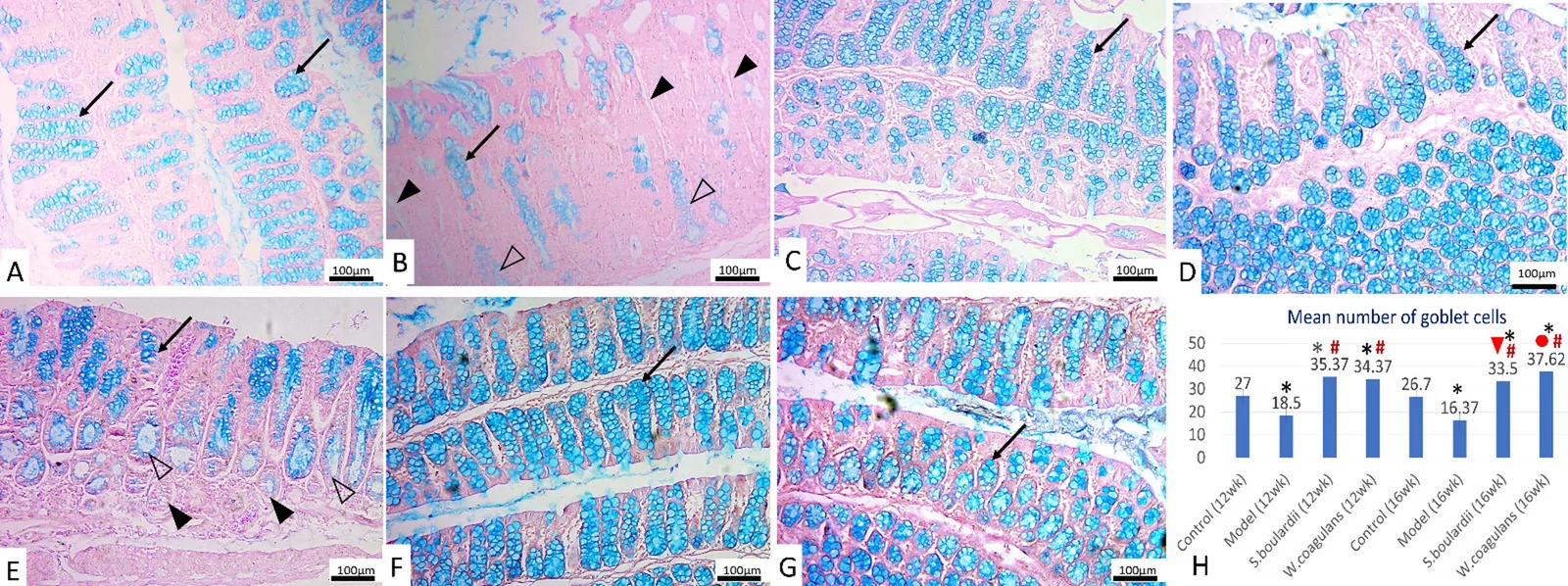
Representative Photomicrographs of the Distal Colon in Various Groups; **A** Group I (Control), **B** Model Group at 12 Weeks, **C** 12-Week S. boulardii- Prophylactic Group, **D** 12-Week W. coagulans- Prophylactic Group, **E** Model Group at 16 Weeks, **F** 16-Week S. boulardii- Prophylactic Group, **G** 16-Week W. coagulans- Prophylactic Group. Key observations include Alcian blue-positive goblet cells (↑); crypts with a complete loss of goblet cells (▲); intact crypts with a reduced number of goblet cells and deficient mucus (∆). **H** *The mean number of goblet cells (± SEM) across different groups: p < 0.05 compared to the control group; #p < 0.05 compared to the Model Groups (12, 16 weeks); ▲p < 0.05 compared to the W. coagulans Groups (12, 16 weeks); ●p < 0.05 compared to the S. boulardii Groups (12, 16 weeks). [Combined Alcian blue–PAS staining x100, scale bar: 100 μm]

**Fig. 6:**
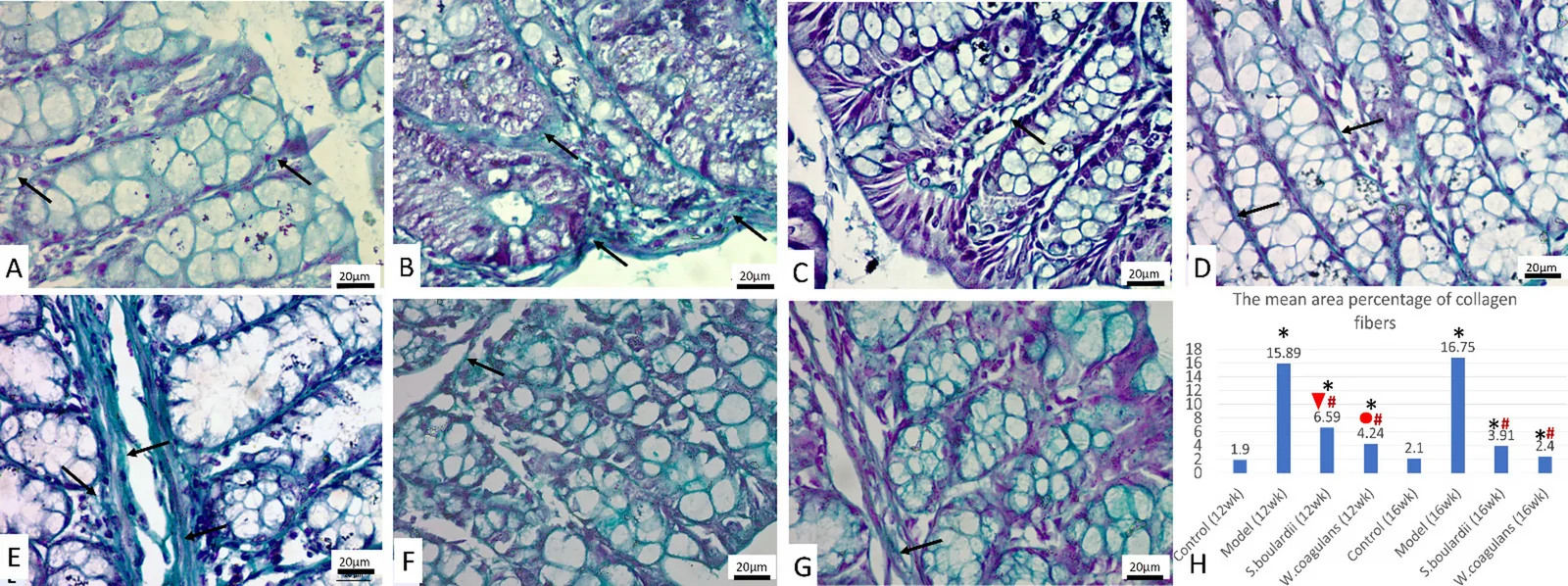
Representative Photomicrographs of the Distal Colon Depicting Collagen Fibers; **A** Group I (Control), **B** Model Group at 12 Weeks, **C** 12-Week S. boulardii- Prophylactic Group, **D** 12-Week W. coagulans- Prophylactic Group, **E** Model Group at 16 Weeks, **F** 16-Week S. boulardii- Prophylactic Group, **G** 16-Week W. coagulans- Prophylactic Group. Collagen fibers (↑) are visible in the lamina propria. **H** *Mean area percentage of collagen fibers (± SEM) across different groups: p < 0.05 compared to the control group; #p < 0.05 compared to Model Groups (12, 16 weeks); ▲p < 0.05 compared to W. coagulans Groups (12, 16 weeks); ●p < 0.05 compared to S. boulardii Groups (12, 16 weeks). [Masson’s trichrome staining x400, scale bar: 20 μm]

Assessment of mucosal secretory integrity and fibrotic changes further supported the histological findings. Alcian blue–PAS staining demonstrated marked goblet cell depletion and reduced mucus content in the AOM/DSS model groups, whereas both *S. boulardii* and *W. coagulans* prophylactic groups showed increased preservation of goblet cell number and size compared with the corresponding model groups (Fig. 5A–G), as supported by histomorphometric analysis (Fig. 5H). Similarly, Masson’s trichrome staining revealed increased collagen deposition in the AOM/DSS model groups, whereas both probiotic groups exhibited reduced collagen accumulation (Fig. 6A–G), confirmed by quantitative histomorphometric assessment (Fig. 6H).

**Fig. 7:**
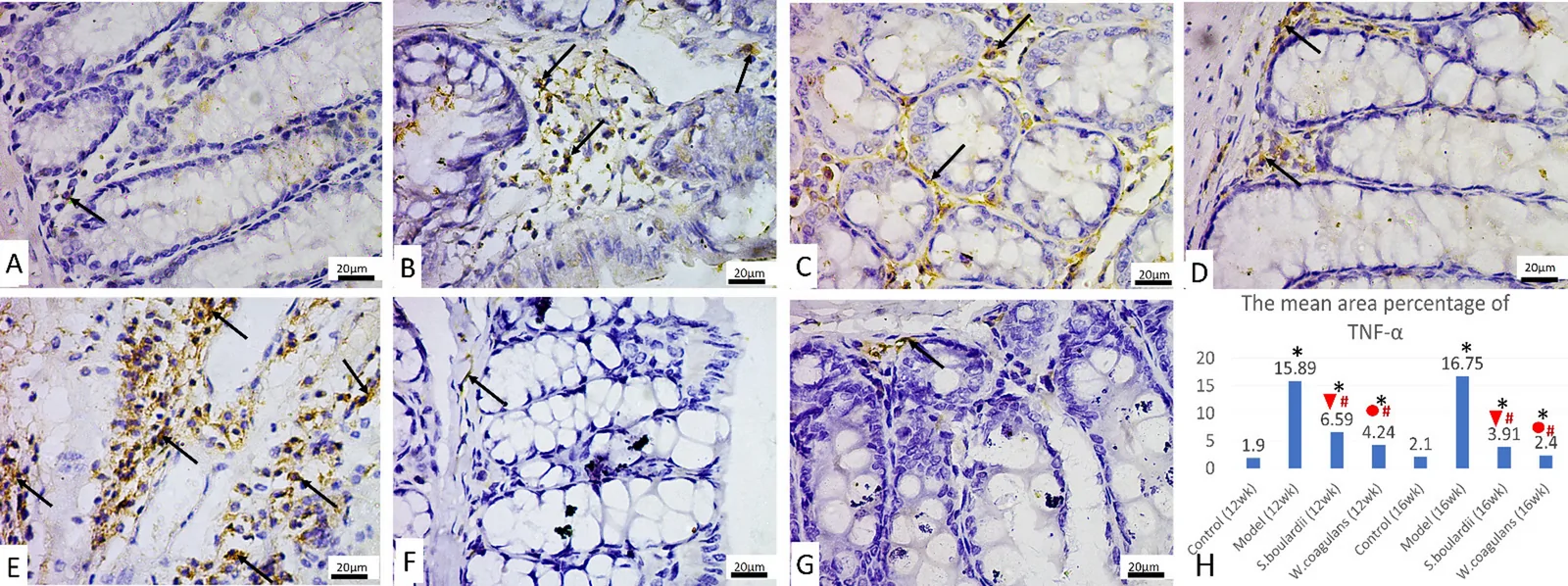
Representative Photomicrographs of Distal Colon Sections from Various Groups. **A** Group I (Control), **B** Model Group at 12 Weeks, **C** 12-Week S. boulardii- Prophylactic Group, **D** 12-Week W. coagulans- Prophylactic Group, **E** Model Group at 16 Weeks, **F** 16-Week S. boulardii- Prophylactic Group, **G** 16-Week W. coagulans- Prophylactic Group. TNF-α cytoplasmic immune reactions are shown in cells of the lamina propria (↑). **H** *Mean area percentage of TNF-α positive reactions (± SEM) across different groups: p < 0.05 compared to the control group; #p < 0.05 compared to Model Groups (12, 16 weeks); ▲p < 0.05 compared to W. coagulans Groups (12, 16 weeks); ●p < 0.05 compared to S. boulardii Groups (12, 16 weeks). Note the increased number and intensity of TNF-α positive cells in the lamina propria of the model groups. [Avidin–biotin peroxidase for TNF-α immunohistochemical staining x400, scale bar: 20 μm]

Consistent with these structural findings, TNF-α immunohistochemical assessment demonstrated increased TNF-α-positive cells in the lamina propria of the AOM/DSS model groups at 12 and 16 weeks compared with controls. Both probiotic prophylactic groups showed reduced TNF-α immunoreactivity compared with the corresponding model groups (Fig. 7A–G), supported by quantitative assessment of the TNF-α-positive area (Fig. 7H).

**Table 2:** Frequency Distribution and Percentage (%) of Histopathological Features in H&E-Stained Distal Colon Sections at 16 Weeks

Groups Finding	Control gp 16 wks	AOM/DSS-Induced Model gp 16 wks	S. boulardii Prophylactic gp 16 wks	W. coagulans Prophylactic gp 16 wks	X^2^	*P*-Value
Dilated Crypts	2 (10%)	7 (35%)	4 (20%)	4 (20%)	3.810	0.283 ^a^
						0.344^b^
						1.000 ^c^
						1.000 ^d^
						1.000 ^e^
						1.000 ^f^
						1.000 ^g^
Obliterated Crypt	0 (0%)	8 (40%)	2 (10%)	1 (5%)	16.337	0.001* ^a^
						0.001* ^b^
						1.000 ^c^
						1.000 ^d^
						0.021* ^e^
						0.005* ^f^
						1.000 ^g^
Areas of ill-defined BM	0 (0%)	17 (85%)	4 (20%)	3 (15%)	40.476	0.000* ^a^
						0.000* ^b^
						0.356 ^c^
						0.932 ^d^
						0.000* ^e^
						0.000* ^f^
						1.000 ^g^
Invasion of colonic-epithelium into lamina-propria	0 (0%)	17 (85%)	3 (15%)	2 (10%)	45.392	0.000* ^a^
						0.000* ^b^
						0.718 ^c^
						1.000 ^d^
						0.000* ^e^
						0.000* ^f^
						1.000 ^g^
Hyperplasia of basal colonic epithelium	1 (5%)	9 (45%)	3 (15%)	2 (10%)	12.718	0.005* ^a^
						0.006* ^b^
						1.000 ^c^
						1.000 ^d^
						0.070 ^e^
						0.021* ^f^
						1.000 ^g^
Dysplasia	0 (0%)	18 (90%)	6 (30%)	5 (25%)	37.809	0.000* ^a^
						0.000* ^b^
						0.059 ^c^
						0.182 ^d^
						0.000* ^e^
						0.000* ^f^
						1.000 ^g^
Increase nuclear-cytoplasmic ratio	0 (0%)	18 (90%)	6 (30%)	5 (25%)	37.809	0.000* ^a^
						0.000* ^b^
						0.059 ^c^
						0.182 ^d^
						0.000* ^e^
						0.000* ^f^
						1.000 ^g^
Irregular nuclear outline	1 (5%)	19 (95%)	5 (25%)	4 (20%)	41.704	0.000* ^a^
						0.000* ^b^
						0.406 ^c^
						1.000 ^d^
						0.000* ^e^
						0.000* ^f^
						1.000 ^g^
Inflammatory cells in submucosa	3 (15%)	10 (50%)	3 (15%)	3 (15%)	10.147	0.017* ^a^
						0.049* ^b^
						1.000 ^c^
						1.000 ^d^
						0.049* ^e^
						0.049* ^f^
						1.000 ^g^
Loss of colonic architecture	0 (0%)	16 (80%)	2 (10%)	2 (10%)	43.733	0.000* ^a^
						0.000* ^b^
						1.000 ^c^
						1.000 ^d^
						0.000* ^e^
						0.000* ^f^
						1.000 ^g^
Sloughing of colonic epithelium	0 (0%)	6 (30%)	0 (0%)	0 (0%)	19.459	0.000* ^a^
						0.001* ^b^
						1.000 ^c^
						1.000 ^d^
						0.001* ^e^
						0.001* ^f^
						1.000 ^g^

Frequency distribution and percentage (%) of histopathological findings in H&E-stained distal colon sections examined at 16 weeks. For each experimental group, four mice were analyzed, with five non-overlapping microscopic fields examined per mouse, resulting in a total of 20 fields per group. Data are expressed as number and percentage (%) of affected fields. Statistical analysis was performed using the Chi-square (χ²) test. Pairwise comparisons were corrected for multiple testing using the Bonferroni method. a: comparison among all groups; b: control vs. AOM/DSS model; c: control vs. *S. boulardii* prophylactic group; d: control vs. *W. coagulans* prophylactic group; e: AOM/DSS model vs. *S. boulardii* prophylactic group; f: AOM/DSS model vs. *W. coagulans* prophylactic group; g: *S. boulardii* vs. *W. coagulans* prophylactic groups.

*p < 0.05 indicates statistical significance; *p* > 0.05 indicates non-significant difference

### Altered miRNA expression profiles in the AOM/DSS-induced model at 16 weeks

**Fig. 8:**
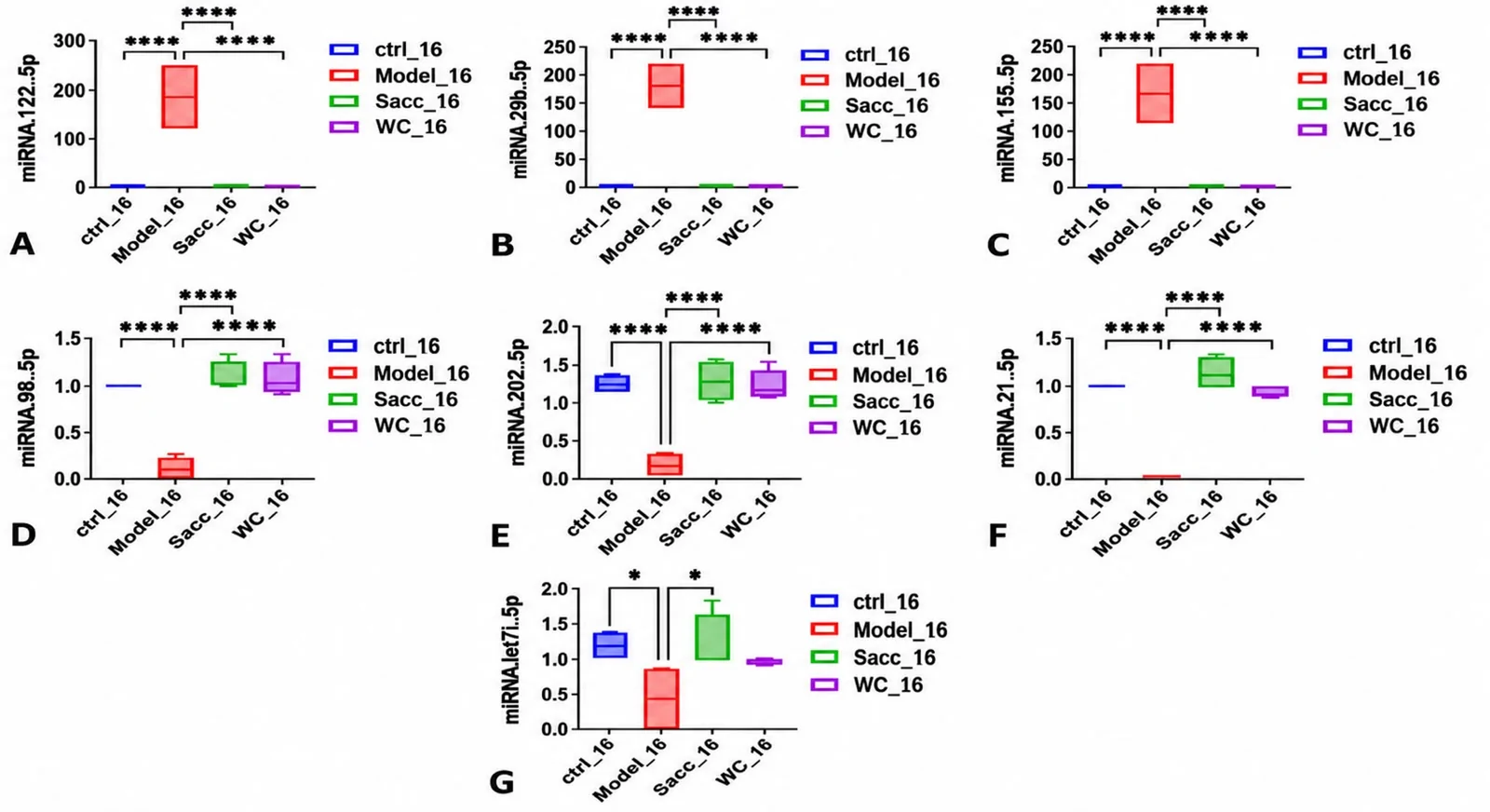
Differential expression of selected miRNAs in distal colon tissues at 16 weeks. Relative expression levels of **A** miRNA122_5p, **B** miRNA29b_5p, **C** miRNA15b_5p, **D** miRNA98_5p, **E** miRNA202_5p, **F** miRNA21_5p, and **G** miRNA_let7i_5p were quantified by qRT-PCR using the ΔCt method and expressed as fold change relative to the control group (ctrl_16). Data are presented as mean ± SD (*n* = 4 mice per group). Statistical analysis was performed using one-way ANOVA followed by Tukey’s multiple comparison test. *p < 0.05, **p < 0.01, ***p < 0.001, ****p < 0.0001

Quantitative real-time PCR analysis was performed on distal colon tissues obtained from 32 mice (*n* = 4 per group). miRNA expression was evaluated only at the 16-week time point, representing the established dysplastic stage of the AOM/DSS model. As shown in Fig. 8 and detailed in Additional Tables S3 and S4, the AOM/DSS model group exhibited marked dysregulation of the investigated miRNAs compared with the control group. Specifically, expression of miR-122-5p increased from 0.927 ± 0.030 in controls to 181.630 ± 72.519, miR-29b-5p from 1.100 ± 0.115 to 185.414 ± 40.948, and miR-15b-5p from 0.962 ± 0.044 to 168.176 ± 61.948 (all *p* < 0.001). Conversely, expression of miR-98-5p, miR-202-5p, miR-21-5p, and let-7i-5p decreased from 0.988 ± 0.014, 1.124 ± 0.143, 0.989 ± 0.012, and 1.131 ± 0.151 in controls to 0.090 ± 0.089, 0.163 ± 0.110, 0.032 ± 0.012, and 0.418 ± 0.480, respectively, in the model group (all *p* < 0.001, except let-7i-5p, *p* = 0.008).

Prophylactic administration of either *Saccharomyces boulardii* or *Weizmannia coagulans* significantly attenuated these alterations (Fig. 8). Relative to the model group, expression of miR-122-5p, miR-29b-5p, and miR-15b-5p decreased to values close to controls (approximately 1.06 ± 0.12, 1.12 ± 0.14, and 1.27 ± 0.24, respectively; all *p* < 0.001 versus model). Likewise, expression of miR-98-5p, miR-202-5p, miR-21-5p, and let-7i-5p increased markedly in both probiotic groups compared with the model group, reaching values comparable to those of controls.

No statistically significant differences were observed between the probiotic groups for most miRNAs, except for miR-21-5p, which was significantly higher in the *W. coagulans* group (*p* = 0.029). Complete relative expression values, ΔCt data, fold changes, confidence intervals, and adjusted *p*-values are provided in Additional Tables S3 and S4.

### Spearman correlation analysis between miRNA expression, cytokine levels, and biochemical parameters at 16 weeks

Significant positive correlations were observed between the pro-inflammatory cytokines IL-6 and IL-1β and the pro-tumorigenic miRNAs miR-122-5p (IL-6: ρ = 0.596, q = 0.032; IL-1β: ρ = 0.745, q = 0.002) and miR-15b-5p (IL-6: ρ = 0.649, q = 0.011; IL-1β: ρ = 0.734, q = 0.003). Conversely, both cytokines were inversely correlated with the anti-inflammatory miR-202-5p (IL-6: ρ = -0.621, q = 0.016; IL-1β: ρ = -0.654, q = 0.011), suggesting a protective regulatory role. These FDR-significant miRNA–cytokine associations are visualized in the correlation network shown in Additional Fig. 13. Additionally, miR-122-5p, miR-29b-5p, and miR-15b-5p showed significant positive correlations with multiple tumor-associated and metabolic markers (CA19-9, CEA, α-FP, liver enzymes, renal indices, glucose, triglycerides, and LDL), and negative correlations with HDL. In contrast, miR-98-5p, miR-202-5p, miR-21-5p, and miR-let-7i-5p were predominantly negatively correlated with inflammatory and metabolic markers while positively associated with HDL.

The complete correlation matrix, including all Spearman’s ρ coefficients, unadjusted p-values, and FDR-adjusted q-values, is provided in Additional Table S5. The effect size analysis demonstrates that probiotic prophylaxis with *S. boulardii* and *W. coagulans* significantly attenuates AOM/DSS-induced inflammation and dysplasia, with large Cohen’s d values (|d| > 1.5) for key outcomes compared to the model group. Both probiotics showed comparable protective effects, with no significant differences between them, and restored most parameters to near-control levels (Additional Table S6).

## Discussion

CRC remains a major global health burden, with its development strongly linked to chronic inflammation and dysregulated immune signaling [31, 32]. Inflammatory pathways, particularly those involving NF-κB, play a central role in driving colorectal dysplasia and tumorigenesis [19]. In this context, probiotics have been proposed as potential modulators of intestinal inflammation and early dysplastic changes, especially in preclinical models of colitis-associated colorectal pathology [33].

**Fig. 9:**
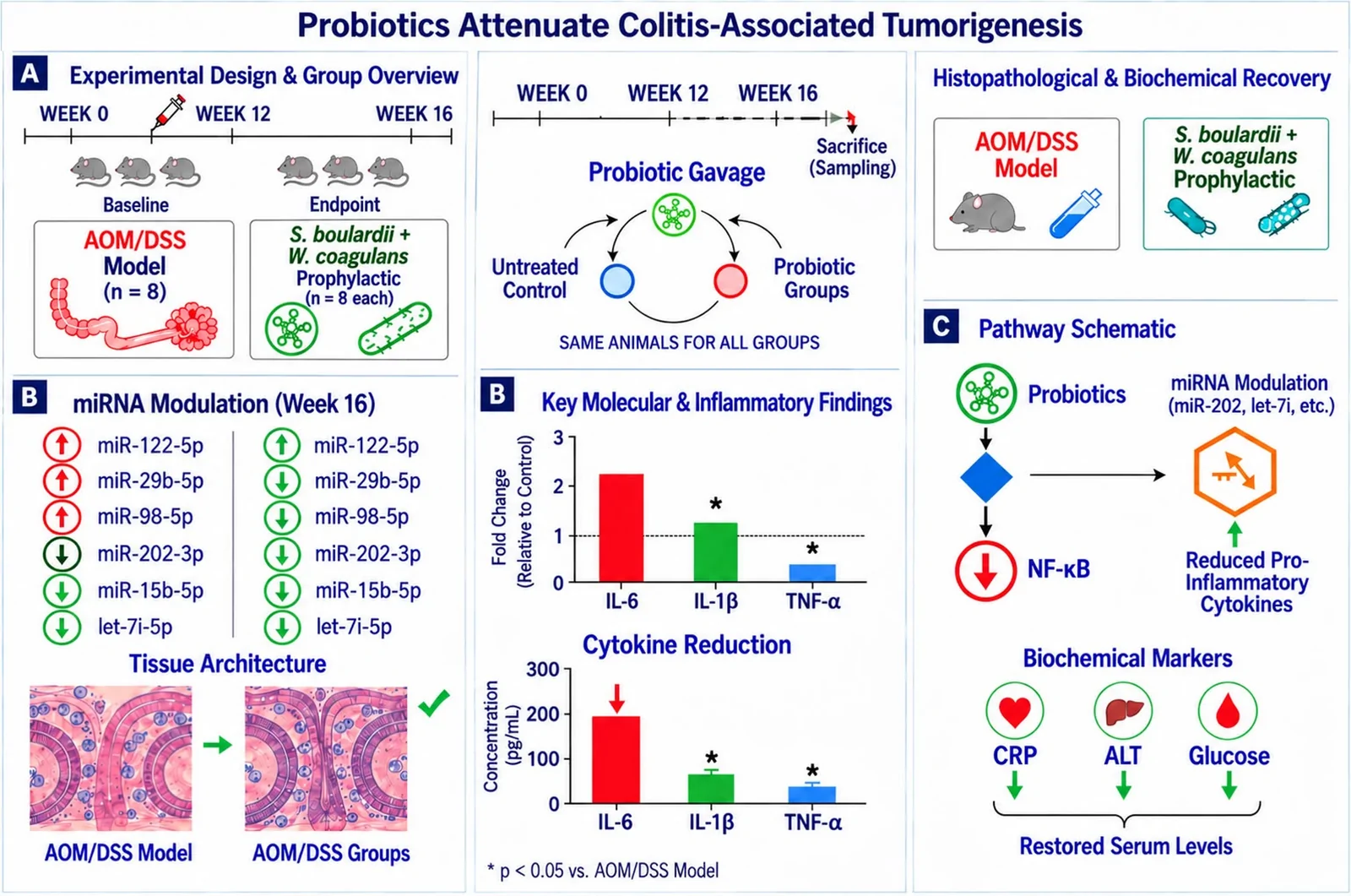
Infographic summary of experimental design, key molecular findings, and histopathological outcomes

In the present study, the AOM/DSS model was used to induce reproducible inflammatory, biochemical, and histopathological alterations consistent with early colorectal dysplasia. Prophylactic administration of *Weizmannia coagulans* and *Saccharomyces boulardii* was associated with reduced pro-inflammatory markers, most likely reflecting indirect effects mediated through attenuation of intestinal inflammation rather than direct molecular or epigenetic targeting. These findings align with previous experimental studies demonstrating protective effects of probiotics in inflammation-driven colorectal models (Fig. 9) [34].

Consistent with prior reports, probiotic prophylaxis was associated with reduced levels of CRP, IL-6, IL-1β, and TNF-α [35, 36]. In the AOM/DSS setting, this anti-inflammatory effect likely contributes to limiting dysplastic progression rather than directly suppressing tumorigenic pathways. Stabilization of body weight in probiotic-treated groups further supports an improvement in systemic inflammatory and metabolic disturbances commonly accompanied by chronic colitis [37].

The similar protective effects observed between *W. coagulans* and *S. boulardii* are noteworthy given the distinct biological characteristics of these microorganisms. *W. coagulans* is a spore-forming bacterium with endospores that provide it with resistance to environmental stress and facilitate survival through the gastrointestinal tract, whereas *S. boulardii* is a probiotic yeast whose eukaryotic cell wall contributes to its interactions with the intestinal mucosa and immune system [38, 39]. These structural differences suggest that the two probiotics likely interact with the host through distinct mechanisms. Indeed, *S. boulardii* has been reported to attenuate NF-κB- and MAPK-mediated inflammatory signaling, whereas *W. coagulans* has demonstrated other biological effects in experimental models, including AMPK/mTOR-mediated autophagy-dependent apoptosis in tumor-bearing mice [40, 41]. Despite these differences, both strains were associated with similar reduction in inflammatory markers and early dysplastic changes, suggesting that the two probiotic species may converge on downstream anti-inflammatory effects. Nevertheless, published evidence directly comparing *W. coagulans* and *S. boulardii* in inflammatory bowel disease or colorectal carcinogenesis remains limited.

Histopathological examinations, especially at 16 weeks, revealed hallmark features of dysplastic changes, such as increased nuclear-to-cytoplasmic ratio, irregular nuclear outlines, and loss of colonic architecture. These findings are consistent with Cuellar-Núñez et al. [42], and Santiago et al. [43] who described similar criteria for dysplasia, including loss of crypt architecture, epithelial hyperplasia, and sloughing. Increased TNF-α expression, observed in our model groups, aligns with the reports of these findings, which were consistent with Aboulthana et al. [44, 45] and Wang et al. [46]. In the current study, increased TNF-α area percentage was noticed in model groups. De Robertis et al. [47] reported that mRNA and protein levels of TNF-α are increased to abnormal levels in the preneoplastic inflamed colonic mucosa. Balkwill [48] reported that inflammation enhances the proliferation of malignant cells, stimulates angiogenesis, and metastasis. Bates and Mercurio [49] reported that TNF-α in the tumor microenvironment may induce the epithelial-mesenchymal transition of malignant cells, which in turn will increase fibrosis. This could explain increased collagen fibers in model groups. These observations are consistent with inflammation-driven dysplasia in experimental models and should not be interpreted as evidence of invasive carcinoma.

In silico analysis identified seven miRNAs (miRNA-202, miRNA-let-7i, miRNA-122, miRNA-98, miRNA-29b, miRNA-21, and miRNA-15b) previously implicated in inflammatory signaling and immune-related pathways accompanied by colorectal carcinogenesis. Dysregulation of these miRNAs in the AOM/DSS model coincided with heightened inflammatory activity, whereas probiotic prophylaxis was associated with partial normalization of their expression, most notably at the 16-week time point. Although NF-κB–related pathways are known to regulate many of these miRNAs [50, 51], they were not directly examined in the present study.

Previous experimental evidence further supports an association between probiotic administration and modulation of host miRNA expression in intestinal disease. Rodríguez-Nogales et al. reported that DSS-induced colitis increased colonic expression of miR-150, miR-155, and miR-223 while reducing miR-143 and miR-375 [52]. Treatment with *Escherichia coli* Nissle 1917 ameliorated intestinal inflammation and significantly reduced the DSS-associated increases in miR-150, miR-155, and miR-223. Although the miRNAs examined differed from those in the present study, a similar overall pattern was observed, as AOM/DSS-induced alterations in the selected miRNAs were generally shifted toward control levels following probiotic prophylaxis. In contrast, Zhang et al. provide evidence involving miR-21-5p, one of the miRNAs examined in the present study [53]. In patients with established colorectal cancer, serum miR-21-5p decreased following radical surgery in both the placebo and probiotic groups, with a significantly greater postoperative reduction in patients receiving Bifidobacterium triplex viable capsules. This differs from the present study, in which AOM/DSS itself reduced colonic miR-21-5p relative to healthy controls, whereas probiotic prophylaxis increased its expression toward control levels, reaching statistical significance in the *W. coagulans* group. The divergent miR-21-5p responses may reflect the markedly different biological and experimental settings, including established human colorectal cancer versus early inflammation-associated dysplasia, circulating versus colonic miRNA expression, perioperative treatment versus prophylaxis, and the use of different probiotic preparations. Taken together, these findings suggest that probiotic-associated miRNA responses may be context-dependent and support interpreting the changes observed in the present study as associated with improvement of the inflammatory environment rather than evidence of direct probiotic regulation of individual miRNAs.

Of note, IL-6 and IL-1β are key mediators of CRC-associated inflammatory signaling, including NF-κB–related pathways [54, 55]. Probiotic prophylaxis was associated with reduced circulating levels of these cytokines, consistent with experimental and clinical evidence demonstrating that *S. boulardii* attenuates pro-inflammatory cytokine expression in inflammatory colorectal conditions [56, 57]. These findings further support an anti-inflammatory role for probiotics in inflammation-driven colorectal dysplasia.

Finally, significant correlations were observed between miRNA expression and biochemical parameters. miRNA-122, miRNA-29b, and miRNA-15b were positively correlated with tumor markers and metabolic indices, while miRNA-98, miRNA-202, miRNA-21, and miRNA-let-7i showed predominantly inverse associations. IL-6 and IL-1β correlated positively with miRNA-122 and miRNA-15b, reinforcing their association with an inflammatory microenvironment. These correlations indicate coordinated changes between miRNA expression and inflammatory or metabolic markers but do not establish causal relationships. The observed normalization of miRNA expression following probiotic administration is likely mediated indirectly through attenuation of intestinal inflammation, rather than via direct epigenetic modulation. This interpretation is supported by the parallel reduction in pro‑inflammatory cytokines (IL‑6, IL‑1β, TNF‑α), which are known regulators of miRNA expression in colitis‑associated dysplasia. While certain probiotic‑derived metabolites or surface molecules could theoretically influence miRNA expression directly, our study design does not permit differentiation between direct and indirect mechanisms. Future studies using germ‑free models, bacterial‑conditioned media, or specific probiotic components are needed to dissect the precise pathways involved.

Although direct validation of mRNA or protein targets for each miRNA was not performed, the coordinated changes in miRNA expression and downstream inflammatory mediators (IL‑6, IL‑1β, TNF‑α) suggest that probiotic prophylaxis correlates with miRNA networks involved in inflammatory signaling during early dysplastic progression. It is important to emphasize that our findings are derived from a controlled, chemically induced mouse model of colitis-associated dysplasia, which does not fully recapitulate the complexity, multifactorial etiology, or chronic progression of human CRC. Therefore, any translational implications for human CRC prevention must be interpreted with caution. Future research should include human biopsy-derived organoids, longitudinal clinical cohorts, and randomized pilot studies to assess whether similar miRNA and inflammatory modulation occurs in humans following probiotic intervention.

## Conclusion

This study supports a potential prophylactic role for *Saccharomyces boulardii* and *Weizmannia coagulans* in attenuating early dysplastic and inflammatory changesin an AOM/DSS-induced mouse model. Probiotic administration was associated with partial normalization of dysregulated miRNA expression and reductions in inflammatory markers, indicating coordinated modulation of inflammatory and molecular responses during early disease stages. While these findings suggest that probiotic prophylaxis may influence pathways linked to inflammation-associated dysplasia, the observed effects are associative and limited to the experimental model. Further studies incorporating microbiota profiling and functional validation are required to clarify underlying mechanisms and long-term protective potential. We recommend that future studies incorporate appropriate vehicle controls, such as a saline or buffer-injected group, to better isolate the effects of the treatment from those associated with injection stress.

## Limitations

Several limitations should be considered when interpreting these findings. First, the absence of direct gut microbiota profiling precludes definitive conclusions regarding probiotic-induced alterations in microbial composition. Additionally, the intestinal survival, recovery, and colonization of the administered probiotic strains were not directly verified. Furthermore, we did not assess whether the administered probiotics (*W. coagulans* and *S. boulardii*) successfully colonized or engrafted in the intestinal tract. Thus, the observed protective effects may be due to transient probiotic presence or indirect modulation of the endogenous microbiota, rather than sustained colonization. Consequently, the observed changes in inflammatory markers, histopathological features, and miRNA expression cannot be directly attributed to microbiota modulation and should be interpreted as associative rather than mechanistic. Further evaluation of additional immunoregulatory and epithelial barrier-related markers, together with mechanistic studies, is warranted to better characterize these interactions and establish their functional relevance in inflammation-associated colorectal dysplasia. Second, the study focused on early dysplastic changes within the AOM/DSS model and did not evaluate long-term progression to invasive colorectal carcinoma. Thirdly, the relatively small sample size per group may limit statistical power despite appropriate post-hoc and multiple-testing corrections. In addition, probiotic-only and individual AOM- or DSS-only control groups were not included, which limits full separation of probiotic-specific effects from carcinogen- or inflammation-induced changes. Furthermore, the use of a single sex may limit the generalizability of the findings, and future studies incorporating both sexes are warranted to assess potential sex-specific responses. Also, it is important to consider that the stress associated with injection procedures may influence the physiological responses observed in our study. This stress could potentially confound the effects of the treatment, making it essential to interpret our findings with caution. Additionally, the study does not distinguish whether miRNA modulation results from direct probiotic–host crosstalk or is secondary to reduced inflammation. Inclusion of probiotic‑only control groups, fecal microbiota transplantation, or in vitro models would help clarify this in future work. Future research should aim to incorporate appropriate vehicle controls to isolate these procedural effects and provide a clearer understanding of the treatment outcomes. These findings are limited to an experimental mouse model and are not directly translatable to humans. Finally, while significant correlations were identified between miRNA expression, inflammatory cytokines, and biochemical parameters, causal relationships and functional validation of miRNA targets were beyond the scope of this study. Future investigations incorporating gut microbiota profiling, SCFA quantification and functional validation of miRNA targets are warranted to elucidate the microbiota–miRNA–inflammation axis.

## Supplementary Information

Supplementary Material 1: Additional Fig. 1: Screenshot from Colon cancer microRNA expression database: Expression of miRNAs; a)miR-202; b)miR-let7i; c)miR-122; d)miR-98; e)miR-29b; f)miR-21; g)miR-15b in different stages of colon cancer tissue in human. Available at: https://www.oncomir.umn.edu/colon/basic_search.php. Additional Fig. 2: Screenshot from Tissue Atlas database: Expression of miRNAs; a)miR-202; b)miR-let7i; c)miR-122; d)miR-98; e)miR-29b; f)miR-21; g)miR-15b in colon cancer tissue in human. Available at: https://ccb-web.cs.uni-saarland.de/tissueatlas/patterns. Additional Fig. 3: Screenshot from dbDEMC: database of differentially expressed miRNAs in Human cancer of miRNAs; a)miR-98; b)miR-29b; c)miR-21; d)miR-15b in colon cancer tissue in human. Available at: http://www.picb.ac.cn/dbDEMC/index.html. Additional Fig. 4: Screenshot from miRò database: miRNA 122 expression in colon cancer tissue in human. Available at: http://microrna.osumc.edu/miro/. Additional Fig. 5: Screenshot from Immune-miR database: miRNA 21 expression in colon cancer tissue in human. Available at:http://biominingbu.org/immunemir/index.html. Additional Fig. 6: Screenshot from DIANA tools-miRpath V.3: The selected miRNAs; miRNA_202, miRNA_let7i, miRNA_122, miRNA_98, miRNA_29b, miRNA_21, miRNA_15b are implicated in cancer and various immunomodulatory pathways. Available at: http://snf-515788.vm.okeanos.grnet.gr/index.php?r=mirpath. Additional Fig. 7: Screenshot from miRWalk database showing the selection of target effector proteins based on interaction with miRNAs; a)miR-122, b)miR-15b, c)miR-29b, d)miR-let7i, e)miR-98b, f)miR-202. Available at: http://mirwalk.umm.uni-heidelberg.de/. Additional Fig. 8: Screenshot from miR database showing the interaction between miR-let7i and IL6 gene. Available at: https://mirdb.org/mirdb/index.html. Additional Fig. 9: Screenshot from Kyoto Encyclopedia of Genes and Genomes database showing selection of IL-6, IL-1B, and TNF-α proteins within the a) DNA sensing, and b) NF-κB signaling pathways which are commonly activated by gut dysbiosis. Available at: https://www.genome.jp/kegg/. Additional Fig. 10: Experimental Design for Evaluating the Effects of Prophylactic Probiotic Strains (*Saccharomyces boulardii* and *Weizmannia coagulans*) in an AOM/DSS-Induced Mouse Model; Thirty-Two male Balb/c mice, aged six weeks and weighing 25–30 grams, were housed under controlled environmental conditions (temperature: 25 ± 3°C, light/dark cycle: 12 hours each). The mice were acclimatized for one week with unrestricted access to food and water. Additional Fig. 11: Experimental Procedure for Sample Collection and Analysis in Mice: Biochemical, Histopathological, and RT-qPCR Evaluations. Additional Fig. 12. Body Weight Dynamics in Control, AOM/DSS model group, and Prophylactic Groups Across the Experimental Timeline.; Body weight measurements are presented as mean ± SD (n = 4 per group). Statistical analysis was performed using one-way ANOVA followed by Tukey’s Multiple Comparison Test. (*P < 0.0001) indicates a significant difference compared to the control group, while (^#^ P < 0.0001) indicates a significant difference compared to the AOM/DSS induced mice group. Additional Fig. 13. Correlation network of significant associations between miRNA expression and pro-inflammatory cytokine levels at week 16. The network depicts significant correlations between miRNA expression levels and the pro-inflammatory cytokines IL-6 and IL-1β, as determined by Spearman correlation analysis. Only correlations that remained significant after false discovery rate (FDR) correction (q < 0.05) are displayed. Solid lines indicate positive correlations, whereas dashed lines indicate negative correlations. Edge labels show the corresponding Spearman correlation coefficients (ρ) and FDR-adjusted q-values.

## Data Availability

No datasets were generated or analysed during the current study.

## Abbreviations

ALT: Alanine Aminotransferase
AOM: Azoxymethane
AST: Aspartate Aminotransferase
CA19.9: Carbohydrate Antigen 19 − 9
CEA: Carcinoembryonic Antigen
CRC: Colorectal Cancer
DSS: Dextran Sulfate Sodium
ELISA: Enzyme-Linked Immunosorbent Assay
H&E: Hematoxylin and Eosin
HDL: High-Density Lipoprotein
IL-1β: Interleukin 1 beta
IL-6: Interleukin 6
LDL: Low-Density Lipoprotein
miRNA: MicroRNA
NF-κB: Nuclear Factor kappa-light-chain-enhancer of activated B cells
p: Probability value (p-value)
PAS: Periodic Acid–Schiff
PCR: Polymerase Chain Reaction
RQ: Relative Quantification
RT-qPCR: Reverse Transcription Quantitative Polymerase Chain Reaction
SD: Standard Deviation
SEM: Standard Error of the Mean
SPSS: Statistical Package for the Social Sciences
TGs: Triglycerides
TNF-α: Tumor Necrosis Factor alpha
