# Supplementary material for: Prophylactic administration of probiotic strains Weizmannia coagulans and Saccharomyces boulardii attenuates early dysplastic changes in an AOM/DSS-induced mouse model
Source: BMC Microbiol. 2026 Oct 6;26:895. doi: 10.1186/s12866-026-05634-z (PMC13675988; doi:10.1186/s12866-026-05634-z)
Supplement: Supplementary file 1 [file 12866_2026_5634_MOESM1_ESM.docx]

**Additional Methods**

I. **miRNA-CRC disease expression:**

In this study we have retrieved seven miRNAs from databases based on their gene expression in CRC. These miRNAs are miRNA 202, miRNA let7i, miRNA 122, miRNA98, miRNA 29b, miRNA21 and miRNA15b.

The 7 miRNAs were selected from public databases; hsa-miR122, hsa-miR29b, hsa-miR 15b, hsa-miR202, hsa-miR 98, hsa-miR 21 and hsa-miR let-7i according to:

1. Their high-ranking score of their differential expression in CRC versus control through the use of more than 2 databases decreasing the false discovery.: A) **Colon cancer microRNA expression database** (1) (Add.Fig.1; a)miR-202; b)miR-let7i; c)miR-122; d)miR-98; e)miR-29b; f)miR-21; g)miR-15b), B) **Tissue atlas database** (2) (Add.Fig.2; a)miR-202; b)miR-let7i; c)miR-122; d)miR-98; e)miR-29b; f)miR-21; g)miR-15b), C) **dbDEMC: database of differentially expressed miRNAs** in cancers (3) (Add.Fig.3; a)miR-98; b)miR-29b; c)miR-21; d)miR-15b), D) **miRo: a miRNA knowledge base** (4) (Add.Fig.4; miR-122),
2. Correlation between the chosen differentially expressed miRNA and immune regulatory mechanisms by **Immune miR database** (5) (Add.Fig.5; miR-21),
3. Then, pathway enrichment analysis of the seven miRNAs was done through **DIANA tools database** for all miRNAs (Add.Fig.6) (6) which showed the implication of the selected miRNAs in CRC and multiple cancers as endometrial, prostate, pancreatic, Renal cell carcinoma and lung cancer. The selected miRNAs were also implicated in immunomodulatory pathways such as Transforming Growth Factor B (TGF-B) signaling pathway, FOXO signaling pathway, P53 signaling pathway, Hippo signaling pathway and AMPK signaling pathway.
II. **Pathway enrichment analysis:**

To contextualize the functional implications of these miRNAs, we performed pathway enrichment analysis using the DIANA tools-miRpath v.3 platform ([DIANA-miRPath v3](http://snf-515788.vm.okeanos.grnet.gr/index.php?r=mirpath)), which allowed us to map the selected miRNAs to known cancer-related, inflammatory, and immunomodulatory pathways. This analysis facilitated the identification of key pathways that may be modulated by the miRNA network, offering insights into the molecular mechanisms of CRC.

The selected miRNAs: miRNA_202, miRNA_let7i, miRNA_122, miRNA_98, miRNA_29b, miRNA_21, miRNA_15b was found to be implicated in cancer, inflammation and various immunomodulatory pathways.

e

d

c

a

b

g

f

**Additional Figure 1:** Screenshot from **Colon cancer microRNA expression database**: Expression of miRNAs; a)miR-202; b)miR-let7i; c)miR-122; d)miR-98; e)miR-29b; f)miR-21; g)miR-15b in different stages of colon cancer tissue in human. Available at: <https>[://www.oncomir.umn.edu/colon/basic_search.php](https://www.oncomir.umn.edu/colon/basic_search.php)

b

a

c

d

e

f

g

**Additional Figure 2:** Screenshot from **Tissue Atlas database**: Expression of miRNAs; a)miR-202; b)miR-let7i; c)miR-122; d)miR-98; e)miR-29b; f)miR-21; g)miR-15b in colon cancer tissue in human. Available at: <https://ccb-web.cs.uni-saarland.de/tissueatlas/patterns>

b

a

d

c

**Additional Figure 3:** Screenshot from **dbDEMC**: database of differentially expressed miRNAs in Human cancer of miRNAs; a)miR-98; b)miR-29b; c)miR-21; d)miR-15b in colon cancer tissue in human. Available at: <http://www.picb.ac.cn/dbDEMC/index.html>

**Additional Figure 4**: Screenshot from **miRò database**: miRNA 122 expression in colon cancer tissue in human. Available at: <http://microrna.osumc.edu/miro/>

**Additional Figure 5:** Screenshot from **Immune-miR database**: miRNA 21 expression in colon cancer tissue in human. Available at:<http://biominingbu.org/immunemir/index.html>

**Additional Figure 6:** Screenshot from **DIANA tools-miRpath V.3**: The selected miRNAs; miRNA_202, miRNA_let7i, miRNA_122, miRNA_98, miRNA_29b, miRNA_21, miRNA_15b are implicated in cancer and various immunomodulatory pathways. Available at: <http://snf-515788.vm.okeanos.grnet.gr/index.php?r=mirpath>

III. **Selection of Target Effector Proteins:**

Building upon the miRNA-pathway associations, particular attention was dedicated to the selection of IL-6, IL-1B, and TNF-α as pivotal effector proteins. This selection is premised on their critical roles within the DNA sensing and NF-κB signaling pathways which are commonly activated by gut dysbiosis and are fundamental to the pathogenesis and advancement of colorectal cancer (CRC). These proteins, integral to cancer development and the modulation of immune responses, serve as key markers in evaluating the immunomodulatory pathways at play in CRC (Add.Figs.7,8,9).

a

b

c

d

e

f

**Additional Figure 7:** Screenshot from **miRWalk database** showing the selection of target effector proteins based on interaction with miRNAs; a)miR-122, b)miR-15b, c)miR-29b, d)miR-let7i, e)miR-98b, f)miR-202. Available at: <http://mirwalk.umm.uni-heidelberg.de/>

**Additional Figure 8:** Screenshot from **miR database** showing the interaction between miR-let7i and IL6 gene. Available at: <https://mirdb.org/mirdb/index.html>

a

b

**Additional Figure 9:** Screenshot from **Kyoto Encyclopedia of Genes and Genomes database** showing selection of IL-6, IL-1B, and TNF-α proteins within the a) DNA sensing, and b) NF-κB signaling pathways which are commonly activated by gut dysbiosis. Available at: <https://www.genome.jp/kegg/>

**Additional Figure 10:** "Experimental Design for Evaluating the Effects of Prophylactic Probiotic Strains (*Saccharomyces boulardii* and *Weizmannia coagulans (formerly Bacillus coagulans)*) in an AOM/DSS-Induced Mouse Model”; Thirty-Two male Balb/c mice, aged six weeks and weighing 25–30 grams, were housed under controlled environmental conditions (temperature: 25 ± 3°C, light/dark cycle: 12 hours each). The mice were acclimatized for one week with unrestricted access to food and water.

**Additional Figure 11:** Experimental Procedure for Sample Collection and Analysis in Mice: Biochemical, Histopathological, and RT-qPCR Evaluations.

**Additional Figure 12.** Body Weight Dynamics in Control, AOM/DSS model group, and Prophylactic Groups Across the Experimental Timeline.; Body weight measurements are presented as mean ± SD (n = 4 per group). Statistical analysis was performed using one-way ANOVA followed by Tukey's Multiple Comparison Test. (*P < 0.0001) indicates a significant difference compared to the control group, while (^#^ P < 0.0001) indicates a significant difference compared to the AOM/DSS induced mice group.

Abbreviations: SB, *Saccharomyces boulardii*; BC, *Weizmannia coagulans (formerly Bacillus coagulans)*

**Additional Figure 13. Correlation network of significant associations between miRNA expression and pro-inflammatory cytokine levels at week 16.** The network depicts significant correlations between miRNA expression levels and the pro-inflammatory cytokines IL-6 and IL-1β, as determined by Spearman correlation analysis. Only correlations that remained significant after false discovery rate (FDR) correction (q < 0.05) are displayed. Solid lines indicate positive correlations, whereas dashed lines indicate negative correlations. Edge labels show the corresponding Spearman correlation coefficients (ρ) and FDR-adjusted q-values.

**Additional Tables**

**Additional Table S1: The criteria that were used to select only those specific genes and miRNA**

| **Criteria** | **Selection Process/Rationale** | **Key Parameters/Examples** |
| --- | --- | --- |
| **1-miRNA-CRC Disease Expression** | - **Database Retrieval:** Seven miRNAs were selected based on their differential expression in colorectal cancer (CRC) from multiple public databases, including: | • Cutoff Criteria: We established a cutoff for selecting miRNAs based on a with thresholds: \|logFC\| > 1, p < 0.05. to ensure that only those with meaningful expression differences were included for validation  Selected miRNAs: hsa-miR-202, hsa-miR-let-7i, hsa-miR-122, hsa-miR-98, hsa-miR-29b, hsa-miR-21, hsa-miR-15b. |
|  | A) Colon Cancer MicroRNA Expression Database. | • High-ranking scores for differential expression across CRC versus control. |
|  | B) Tissue Atlas Database. | • Examples: miR-202, miR-let-7i, miR-122, miR-98, miR-29b, miR-21, miR-15b. |
|  | C) dbDEMC: Database of Differentially Expressed miRNAs in Cancers. | • Consistent expression patterns across multiple datasets to reduce false discovery. |
|  | D) miRo: A miRNA Knowledge Base . |  |
|  | - **Correlation with Immune Mechanisms:** Used the Immune miR database to assess the relationship between selected miRNAs and immune regulatory mechanisms. | • Example: miR-21 linked to immune modulation. |
| **2-Pathway Enrichment Analysis** | - **Functional Implications:** Performed pathway enrichment analysis using DIANA tools-miRpath v.3 platform to map selected miRNAs to cancer-related, inflammatory, and immunomodulatory pathways. | • Identified key pathways modulated by the miRNA network, providing insights into the molecular mechanisms of CRC.  • Implicated pathways: TGF-β signaling, FOXO signaling, P53 signaling, Hippo signaling, AMPK signaling. |
|  | - **Selected Pathways:** The seven miRNAs were found to be implicated in cancer, inflammation, and various immunomodulatory pathways. | • Examples: Pathways associated with immune response and tumor progression, highlighting the relevance of these miRNAs in CRC. |
| **3-miRNA-mRNA Interaction:** | miRNA-mRNA Interaction: Predicted interactions were assessed using mirWalk, identifying miRNAs that target relevant mRNAs involved in colorectal cancer pathways. | Examples: miR-21 targets include genes related to cell proliferation and inflammation, such as PTEN and IL-6 |
| **4-mRNA targets relation to gut dysbiosis** | Kyoto Encyclopedia of Genes and Genomes database | Selection of IL-6, IL-1B, and TNF-α proteins within the a) DNA sensing, and b) NF-κB signaling pathways which are commonly activated by gut dysbiosis |
| **5-Considered but Excluded** | - Excluded miRNAs with inconsistent expression across datasets or those lacking functional annotations in colorectal cancer pathways. | • Example exclusions: miRNAs not linked to inflammatory responses or those with minimal empirical support in the literature. |
| **6-Previous Literature Validation** | - Selected miRNAs were validated by prior studies demonstrating their roles in colorectal cancer and response to probiotics. | • References: Supporting studies for miR-21 in inflammation and miR-122 in tumor suppression, highlighting their relevance to our findings. |

**Additional Table S2: Experimental Design and Sample Allocation**

| Group | Total Mice (n=32) | Subgroup (Sacrifice Week) | Mice per Subgroup | Histology & IHC (W12 + W16) | miRNA‑qPCR (W12 + W16) | ELISA & Biochemistry (W12 + W16) | Consistent Animal Use Across Assays? |
| --- | --- | --- | --- | --- | --- | --- | --- |
| **Control** | 8 | Week 12 | 4 | 4 | 4 | 4 | ✅ Yes – same 4 animals |
|  |  | Week 16 | 4 | 4 | 4 | 4 | ✅ Yes – same 4 animals |
| **AOM/DSS Model** | 8 | Week 12 | 4 | 4 | 4 | 4 | ✅ Yes – same 4 animals |
|  |  | Week 16 | 4 | 4 | 4 | 4 | ✅ Yes – same 4 animals |
| ***S. boulardii* Prophylactic** | 8 | Week 12 | 4 | 4 | 4 | 4 | ✅ Yes – same 4 animals |
|  |  | Week 16 | 4 | 4 | 4 | 4 | ✅ Yes – same 4 animals |
| ***W. coagulans* Prophylactic** | 8 | Week 12 | 4 | 4 | 4 | 4 | ✅ Yes – same 4 animals |
|  |  | Week 16 | 4 | 4 | 4 | 4 | ✅ Yes – same 4 animals |
| **Total Mice (All Groups)** | **32** | **Week 12 Total** | **16** | **16** |  |  |  |

**Additional Table S3: The effect of AOM/DSS induced dysplastic changes and prophylactic probiotics administration on the different biochemical parameters in 16 weeks**

| **Groups**  **Biochemical parameters** | **Control gp**  **16 wks** | **AOM/DSS-Induced Model gp**  **16 wks** | ***S. boulardii* Prophylactic gp**  **16 wks** | ***W. coagulans* Prophylactic gp**  **16 wks** | **F** | **P-Value** |
| --- | --- | --- | --- | --- | --- | --- |
|  | Mean ± SD | Mean ± SD | Mean ± SD | Mean ± SD |  |  |
| **Albumin (g/dl)** | 3.42 ± 0.33 | 2.97 ± 0.26 | 3.55 ± 0.12 | 3.65 ± 0.12 | 6.709 | 0.006*^a^ |
|  |  |  |  |  |  | 0.070^b^ |
|  |  |  |  |  |  | 0.867^c^ |
|  |  |  |  |  |  | 0.532^d^ |
|  |  |  |  |  |  | 0.018*^e^ |
|  |  |  |  |  |  | 0.006*^f^ |
|  |  |  |  |  |  | 0.925^g^ |
| **Total Protein (g/dl)** | 5.60±0.21 | 4.72±0.57 | 5.32±0.25 | 5.32±0.27 | 4.24 | 0.029*^a^ |
|  |  |  |  |  |  | 0.021*^b^ |
|  |  |  |  |  |  | 0.704^c^ |
|  |  |  |  |  |  | 0.704^d^ |
|  |  |  |  |  |  | 0.137^e^ |
|  |  |  |  |  |  | 0.137^f^ |
|  |  |  |  |  |  | 0.999^g^ |
| **CRP (ng/mL)** | 20.50±2.64 | 45.00±2.94 | 31.00±1.82 | 23.25±4.03 | 54.74 | 0.000*^a^ |
|  |  |  |  |  |  | 0.000*^b^ |
|  |  |  |  |  |  | 0.001*^c^ |
|  |  |  |  |  |  | 0.574^d^ |
|  |  |  |  |  |  | 0.000*^e^ |
|  |  |  |  |  |  | 0.000*^f^ |
|  |  |  |  |  |  | 0.014*^g^ |
| **CA19.9 (U/mL)** | 34.25±10.69 | 109.30±50.93 | 57.50±6.45 | 58.25±5.90 | 5.755 | 0.011*^a^ |
|  |  |  |  |  |  | 0.008*^b^ |
|  |  |  |  |  |  | 0.611^c^ |
|  |  |  |  |  |  | 0.588^d^ |
|  |  |  |  |  |  | 0.069^e^ |
|  |  |  |  |  |  | 0.074^f^ |
|  |  |  |  |  |  | 0.999^g^ |
| **CEA (U/mL)** | 19.50 ± 4.04 | 105.0 ± 13.52 | 48.75 ± 5.37 | 44.25 ± 6.44 | 77.4 | 0.000*^a^ |
|  |  |  |  |  |  | 0.000*^b^ |
|  |  |  |  |  |  | 0.001*^c^ |
|  |  |  |  |  |  | 0.005*^d^ |
|  |  |  |  |  |  | 0.000*^e^ |
|  |  |  |  |  |  | 0.000*^f^ |
|  |  |  |  |  |  | 0.864^g^ |
| **α-FP (ng/mL)** | 9.00 ± 2.58 | 64.50 ± 10.25 | 34.50 ± 9.74 | 32.75 ± 8.18 | 30.24 | 0.000*^a^ |
|  |  |  |  |  |  | 0.000*^b^ |
|  |  |  |  |  |  | 0.004*^c^ |
|  |  |  |  |  |  | 0.007*^d^ |
|  |  |  |  |  |  | 0.001*^e^ |
|  |  |  |  |  |  | 0.000*^f^ |
|  |  |  |  |  |  | 0.990^g^ |
| **ALT (U/L)** | 52.00±21.79 | 105.5±16.46 | 53.25±9.35 | 43.00±7.43 | 14.53 | 0.000*^a^ |
|  |  |  |  |  |  | 0.001*^b^ |
|  |  |  |  |  |  | 0.999^c^ |
|  |  |  |  |  |  | 0.827^d^ |
|  |  |  |  |  |  | 0.001*^e^ |
|  |  |  |  |  |  | 0.000*^f^ |
|  |  |  |  |  |  | 0.767^g^ |
| **AST (U/L)** | 59.75±9.53 | 142.8±4.99 | 73.75±6.85 | 64.75±9.28 | 96.58 | 0.000*^a^ |
|  |  |  |  |  |  | 0.000*^b^ |
|  |  |  |  |  |  | 0.108^c^ |
|  |  |  |  |  |  | 0.807^d^ |
|  |  |  |  |  |  | 0.000*^e^ |
|  |  |  |  |  |  | 0.000*^f^ |
|  |  |  |  |  |  | 0.407^g^ |
| **Creatinine (mg/dL)** | 0.89±0.07 | 2.50±0.62 | 1.14±0.13 | 1.08±0.13 | 20.28 | 0.000^a^ |
|  |  |  |  |  |  | 0.000*^b^ |
|  |  |  |  |  |  | 0.695^c^ |
|  |  |  |  |  |  | 0.838^d^ |
|  |  |  |  |  |  | 0.000*^e^ |
|  |  |  |  |  |  | 0.000*^f^ |
|  |  |  |  |  |  | 0.992^g^ |
| **Urea (mg/dL)** | 43.00±4.24 | 63.25±8.99 | 45.75±4.34 | 54.75±7.32 | 7.896 | 0.003*^a^ |
|  |  |  |  |  |  | 0.004*^b^ |
|  |  |  |  |  |  | 0.931^c^ |
|  |  |  |  |  |  | 0.103^d^ |
|  |  |  |  |  |  | 0.012*^e^ |
|  |  |  |  |  |  | 0.304^f^ |
|  |  |  |  |  |  | 0.261^g^ |
| **Glucose (mg/dL)** | 125.0±12.41 | 218.0±31.40 | 163.0±10.86 | 171.5±14.84 | 15.8 | 0.000*^a^ |
|  |  |  |  |  |  | 0.000*^b^ |
|  |  |  |  |  |  | 0.067^c^ |
|  |  |  |  |  |  | 0.022*^d^ |
|  |  |  |  |  |  | 0.007*^e^ |
|  |  |  |  |  |  | 0.022*^f^ |
|  |  |  |  |  |  | 0.922^g^ |
| **Triglycerides (mg/dL)** | 122.5±6.45 | 211.3±10.66 | 149.8±14.73 | 155.3±14.86 | 37.41 | 0.000*^a^ |
|  |  |  |  |  |  | 0.000*^b^ |
|  |  |  |  |  |  | 0.035*^c^ |
|  |  |  |  |  |  | 0.011*^d^ |
|  |  |  |  |  |  | 0.000*^e^ |
|  |  |  |  |  |  | 0.000*^f^ |
|  |  |  |  |  |  | 0.917^g^ |
| **HDL (mg/dL)** | 42.50±3.69 | 37.50±3.10 | 38.55±4.72 | 43.75±1.70 | 3.005 | 0.072^a^ |
|  |  |  |  |  |  | 0.230^b^ |
|  |  |  |  |  |  | 0.412^c^ |
|  |  |  |  |  |  | 0.955^d^ |
|  |  |  |  |  |  | 0.972^e^ |
|  |  |  |  |  |  | 0.103^f^ |
|  |  |  |  |  |  | 0.204^g^ |
| **LDL (mg/dL)** | 69.00±4.08 | 155.8±4.34 | 95.00±13.19 | 90.00±1.41 | 105.1 | 0.000*^a^ |
|  |  |  |  |  |  | 0.000*^b^ |
|  |  |  |  |  |  | 0.001*^c^ |
|  |  |  |  |  |  | 0.007*^d^ |
|  |  |  |  |  |  | 0.000*^e^ |
|  |  |  |  |  |  | 0.000*^f^ |
|  |  |  |  |  |  | 0.767^g^ |

Data are presented as mean ± SD. Statistical analysis was performed using one-way ANOVA; Tukey’s post-hoc comparisons included overall group comparison (a), control vs model (b), control vs *S. boulardii* (c), control vs *W. coagulans* (d), model vs *S. boulardii* (e), model vs *B. coagulans* (f), and *S. boulardii* vs *W. coagulans* (g). A p-value < 0.05 was considered statistically significant.

**Additional Table S4: The Effect of AOM/DSS-induced dysplastic changes and prophylactic probiotic administration on the expression profile of the selected miRNA panel at 16 weeks.**

| **Groups**  **Biomarkers** | **Control gp**  **16 wks** | **AOM/DSS-Induced Model gp**  **16 wks** | ***S. boulardii* Prophylactic gp**  **16 wks** | ***W. coagulans* Prophylactic gp**  **16 wks** | **F** | **p‐value** |
| --- | --- | --- | --- | --- | --- | --- |
|  | mean ± SD  95% CI | mean ± SD  95% CI | mean ± SD  95% CI | mean ± SD  95% CI |  |  |
| miRNA122_5p | 0.927±0.030  (0.88-0.97) | 181.630±72.519  (66.23-297.02) | 1.064±0.123  (0.87-1.25) | 1.060±0.066  (0.95-1.16) | 24.812 | 0.000* **^a^** |
|  |  |  |  |  |  | 0.000* **^b^** |
|  |  |  |  |  |  | 0.996 **^c^** |
|  |  |  |  |  |  | 0.996 **^d^** |
|  |  |  |  |  |  | 0.000* **^e^** |
|  |  |  |  |  |  | 0.000* **^f^** |
|  |  |  |  |  |  | 1.000 **^g^** |
| miRNA29b_5p | 1.100±0.115  (0.91-1.28) | 185.414±40.948  (120.25-250.57) | 1.120±0.164  (0.86-1.38) | 0.984±0.062  (0.88-1.08) | 81.069 | 0.000* **^a^** |
|  |  |  |  |  |  | 0.000* **^b^** |
|  |  |  |  |  |  | 0.999 **^c^** |
|  |  |  |  |  |  | 0.994 **^d^** |
|  |  |  |  |  |  | 0.000* **^e^** |
|  |  |  |  |  |  | 0.000* **^f^** |
|  |  |  |  |  |  | 0.993 **^g^** |
| miRNA15b_5p | 0.962  ±0.044  (0.89-1.03) | 168.176±61.948  (69.60-266.75) | 1.265±0.242  (0.88-1.65) | 1.000 ±0.019  (0.99-0.97) | 29.105 | 0.000* **^a^** |
|  |  |  |  |  |  | 0.000* **^b^** |
|  |  |  |  |  |  | 0.989 **^c^** |
|  |  |  |  |  |  | 0.999 **^d^** |
|  |  |  |  |  |  | 0.000* **^e^** |
|  |  |  |  |  |  | 0.000* **^f^** |
|  |  |  |  |  |  | 0.991 **^g^** |
| miRNA98_5p | 0.988 ±0.014  (0.97-1.00) | 0.090 ±0.089  (-0.05-0.23) | 1.048±0.144  (0.82-1.27) | 1.033±0.164  (0.77-1.29) | 62.546 | 0.000* **^a^** |
|  |  |  |  |  |  | 0.000* **^b^** |
|  |  |  |  |  |  | 0.989 **^c^** |
|  |  |  |  |  |  | 0.999 **^d^** |
|  |  |  |  |  |  | 0.000* **^e^** |
|  |  |  |  |  |  | 0.000* **^f^** |
|  |  |  |  |  |  | 0.991 **^g^** |
| miRNA202_5p | 1.124±0.143  (0.89-1.35) | 0.163±0.110  (-0.01-0.33) | 1.174±0.254  (0.77-1.57) | 1.107±0.182  (0.82-1.39) | 29.127 | 0.000* **^a^** |
|  |  |  |  |  |  | 0.000* **^b^** |
|  |  |  |  |  |  | 0.701 **^c^** |
|  |  |  |  |  |  | 0.894 **^d^** |
|  |  |  |  |  |  | 0.000* **^e^** |
|  |  |  |  |  |  | 0.000* **^f^** |
|  |  |  |  |  |  | 0.606 **^g^** |
| miRNA21_5p | 0.989±0.012  (0.97-1.00) | 0.032±0.012  (0.01-0.05) | 1.157±0.186  (0.86-1.45) | 0.987±0.051  (0.91-1.06) | 111.976 | 0.000* **^a^** |
|  |  |  |  |  |  | 0.000* **^b^** |
|  |  |  |  |  |  | 0.031* **^c^** |
|  |  |  |  |  |  | 0.976 **^d^** |
|  |  |  |  |  |  | 0.000* **^e^** |
|  |  |  |  |  |  | 0.000* **^f^** |
|  |  |  |  |  |  | 0.029* **^g^** |
| miRNA_let7i_5p | 1.131±0.151  (0.89-1.37) | 0.418±0.480  (-0.34-1.18) | 1.183±0.395  (0.55-1.81) | 0.967±0.038  (0.91-1.02) | 4.788 | 0.020* **^a^** |
|  |  |  |  |  |  | 0.008* **^b^** |
|  |  |  |  |  |  | 0.820 **^c^** |
|  |  |  |  |  |  | 0.485 **^d^** |
|  |  |  |  |  |  | 0.005* **^e^** |
|  |  |  |  |  |  | 0.032* **^f^** |
|  |  |  |  |  |  | 0.359 **^g^** |

**Additional Table S4.** Effect of AOM/DSS-induced dysplastic changes and prophylactic probiotic administration on the expression profile of the selected miRNA panel at 16 weeks. Data are presented as mean ± SD with corresponding 95% confidence intervals (CI). miRNA expression levels were quantified by RT-qPCR and normalized to the appropriate endogenous control using the 2^−ΔΔCt method. Statistical analysis was performed using one-way ANOVA followed by Tukey’s post-hoc multiple comparison test. Overall group differences are indicated by (a), while pairwise comparisons are denoted as follows: control vs. model (b), control vs. S. boulardii (c), control vs. W. coagulans (d), model vs. S. boulardii (e), model vs. W. coagulans (f), and S. boulardii vs. W. coagulans (g).
A p-value < 0.05 was considered statistically significant. * indicates statistically significant differences.

**Additional Table S5: Spearman Correlation Matrix of miRNA Expression, Cytokine Levels, and Biochemical Parameters (16‑Week Time Point, n = 16 mice)**
*Correlations are considered significant at* ***q < 0.05*** *after Benjamini–Hochberg FDR correction.*

| **Variable** | **miRNA122_5p (ρ / q)** | **miRNA29b_5p (ρ / q)** | **miRNA15b_5p (ρ / q)** | **miRNA98_5p (ρ / q)** | **miRNA202_5p (ρ / q)** | **miRNA21_5p (ρ / q)** | **miRNA_let7i_5p (ρ / q)** | **IL‑6 (ρ / q)** | **IL‑1β (ρ / q)** | **CA19.9 (ρ / q)** | **CEA (ρ / q)** | **α‑FP (ρ / q)** | **ALT (ρ / q)** | **AST (ρ / q)** | **Creatinine (ρ / q)** | **Urea (ρ / q)** | **Glucose (ρ / q)** | **Triglycerides (ρ / q)** | **HDL (ρ / q)** | **LDL (ρ / q)** |
| --- | --- | --- | --- | --- | --- | --- | --- | --- | --- | --- | --- | --- | --- | --- | --- | --- | --- | --- | --- | --- |
| **miRNA122_5p** | 1.000 | 0.584 / 0.035 | 0.792 / 0.001 | −0.410 / 0.145 | −0.460 / 0.097 | −0.545 / 0.049 | −0.718 / 0.005 | 0.596 / 0.032 | 0.745 / 0.002 | 0.737 / 0.004 | 0.766 / 0.003 | 0.822 / 0.001 | 0.446 / 0.107 | 0.721 / 0.004 | 0.820 / 0.001 | 0.694 / 0.006 | 0.833 / 0.001 | 0.920 / 0.001 | −0.229 / 0.414 | 0.907 / 0.001 |
| **miRNA29b_5p** | 0.584 / 0.035 | 1.000 | 0.589 / 0.034 | −0.514 / 0.058 | −0.394 / 0.159 | −0.743 / 0.003 | −0.522 / 0.055 | 0.679 / 0.007 | 0.467 / 0.089 | 0.371 / 0.181 | 0.635 / 0.013 | 0.459 / 0.096 | 0.657 / 0.011 | 0.634 / 0.013 | 0.604 / 0.024 | 0.362 / 0.183 | 0.515 / 0.058 | 0.486 / 0.071 | −0.329 / 0.235 | 0.520 / 0.056 |
| **miRNA15b_5p** | 0.792 / 0.001 | 0.589 / 0.034 | 1.000 | −0.451 / 0.101 | −0.632 / 0.014 | −0.413 / 0.140 | −0.589 / 0.034 | 0.649 / 0.011 | 0.734 / 0.003 | 0.686 / 0.008 | 0.711 / 0.007 | 0.824 / 0.001 | 0.505 / 0.064 | 0.845 / 0.001 | 0.692 / 0.009 | 0.628 / 0.014 | 0.775 / 0.001 | 0.832 / 0.001 | −0.355 / 0.197 | 0.866 / 0.001 |
| **miRNA98_5p** | −0.410 / 0.145 | −0.514 / 0.058 | −0.451 / 0.101 | 1.000 | 0.668 / 0.010 | 0.677 / 0.009 | 0.634 / 0.013 | −0.452 / 0.100 | −0.400 / 0.147 | −0.328 / 0.235 | −0.614 / 0.018 | −0.617 / 0.017 | −0.620 / 0.016 | −0.647 / 0.012 | −0.402 / 0.146 | −0.522 / 0.055 | −0.502 / 0.065 | −0.439 / 0.109 | 0.523 / 0.054 | −0.485 / 0.073 |
| **miRNA202_5p** | −0.460 / 0.097 | −0.394 / 0.159 | −0.632 / 0.014 | 0.668 / 0.010 | 1.000 | 0.395 / 0.158 | 0.527 / 0.052 | −0.621 / 0.016 | −0.654 / 0.011 | −0.591 / 0.022 | −0.592 / 0.022 | −0.690 / 0.006 | −0.524 / 0.054 | −0.599 / 0.021 | −0.613 / 0.018 | −0.535 / 0.050 | −0.709 / 0.005 | −0.448 / 0.104 | 0.570 / 0.034 | −0.668 / 0.010 |
| **miRNA21_5p** | −0.545 / 0.049 | −0.743 / 0.003 | −0.413 / 0.140 | 0.677 / 0.009 | 0.395 / 0.158 | 1.000 | 0.561 / 0.040 | −0.496 / 0.069 | −0.356 / 0.196 | −0.186 / 0.507 | −0.386 / 0.161 | −0.370 / 0.181 | −0.576 / 0.033 | −0.522 / 0.055 | −0.311 / 0.259 | −0.474 / 0.079 | −0.394 / 0.159 | −0.442 / 0.113 | 0.029 / 0.914 | −0.405 / 0.143 |
| **miRNA_let7i_5p** | −0.718 / 0.005 | −0.522 / 0.055 | −0.589 / 0.034 | 0.634 / 0.013 | 0.527 / 0.052 | 0.561 / 0.040 | 1.000 | −0.474 / 0.079 | −0.476 / 0.077 | −0.694 / 0.009 | −0.708 / 0.007 | −0.726 / 0.005 | −0.461 / 0.097 | −0.624 / 0.015 | −0.656 / 0.011 | −0.810 / 0.001 | −0.706 / 0.007 | −0.712 / 0.006 | 0.282 / 0.310 | −0.635 / 0.013 |
| **IL‑6** | 0.596 / 0.032 | 0.679 / 0.007 | 0.649 / 0.011 | −0.452 / 0.100 | −0.621 / 0.016 | −0.496 / 0.069 | −0.474 / 0.079 | 1.000 | 0.542 / 0.047 | 0.559 / 0.040 | 0.626 / 0.014 | 0.574 / 0.035 | 0.671 / 0.008 | 0.702 / 0.006 | 0.713 / 0.006 | 0.367 / 0.184 | 0.589 / 0.034 | 0.541 / 0.048 | −0.400 / 0.147 | 0.601 / 0.031 |
| **IL‑1β** | 0.745 / 0.002 | 0.467 / 0.089 | 0.734 / 0.003 | −0.400 / 0.147 | −0.654 / 0.011 | −0.356 / 0.196 | −0.476 / 0.077 | 0.542 / 0.047 | 1.000 | 0.586 / 0.034 | 0.692 / 0.009 | 0.685 / 0.009 | 0.660 / 0.010 | 0.664 / 0.010 | 0.789 / 0.002 | 0.513 / 0.058 | 0.760 / 0.003 | 0.792 / 0.002 | −0.516 / 0.057 | 0.760 / 0.003 |
| **CA19.9** | 0.737 / 0.004 | 0.371 / 0.181 | 0.686 / 0.008 | −0.328 / 0.235 | −0.591 / 0.022 | −0.186 / 0.507 | −0.694 / 0.009 | 0.559 / 0.040 | 0.586 / 0.034 | 1.000 | 0.726 / 0.005 | 0.715 / 0.006 | 0.343 / 0.212 | 0.677 / 0.009 | 0.753 / 0.004 | 0.646 / 0.012 | 0.807 / 0.002 | 0.638 / 0.013 | −0.294 / 0.289 | 0.784 / 0.002 |
| **CEA** | 0.766 / 0.003 | 0.635 / 0.013 | 0.711 / 0.007 | −0.614 / 0.018 | −0.592 / 0.022 | −0.386 / 0.161 | −0.708 / 0.007 | 0.626 / 0.014 | 0.692 / 0.009 | 0.726 / 0.005 | 1.000 | 0.803 / 0.002 | 0.636 / 0.013 | 0.820 / 0.001 | 0.838 / 0.001 | 0.710 / 0.007 | 0.835 / 0.001 | 0.797 / 0.002 | −0.507 / 0.062 | 0.806 / 0.002 |
| **α‑FP** | 0.822 / 0.001 | 0.459 / 0.096 | 0.824 / 0.001 | −0.617 / 0.017 | −0.690 / 0.006 | −0.370 / 0.181 | −0.726 / 0.005 | 0.574 / 0.035 | 0.685 / 0.009 | 0.715 / 0.006 | 0.803 / 0.002 | 1.000 | 0.355 / 0.197 | 0.723 / 0.005 | 0.790 / 0.002 | 0.710 / 0.007 | 0.850 / 0.001 | 0.832 / 0.001 | −0.523 / 0.054 | 0.913 / 0.001 |
| **ALT** | 0.446 / 0.107 | 0.657 / 0.011 | 0.505 / 0.064 | −0.620 / 0.016 | −0.524 / 0.054 | −0.576 / 0.033 | −0.461 / 0.097 | 0.671 / 0.008 | 0.660 / 0.010 | 0.343 / 0.212 | 0.636 / 0.013 | 0.355 / 0.197 | 1.000 | 0.752 / 0.004 | 0.542 / 0.048 | 0.337 / 0.218 | 0.483 / 0.073 | 0.517 / 0.057 | −0.502 / 0.065 | 0.410 / 0.145 |
| **AST** | 0.721 / 0.004 | 0.634 / 0.013 | 0.845 / 0.001 | −0.647 / 0.012 | −0.599 / 0.021 | −0.522 / 0.055 | −0.624 / 0.015 | 0.702 / 0.006 | 0.664 / 0.010 | 0.677 / 0.009 | 0.820 / 0.001 | 0.723 / 0.005 | 0.752 / 0.004 | 1.000 | 0.629 / 0.014 | 0.634 / 0.013 | 0.794 / 0.002 | 0.764 / 0.003 | −0.380 / 0.166 | 0.742 / 0.004 |
| **Creatinine** | 0.820 / 0.001 | 0.604 / 0.024 | 0.692 / 0.009 | −0.402 / 0.146 | −0.613 / 0.018 | −0.311 / 0.259 | −0.656 / 0.011 | 0.713 / 0.006 | 0.789 / 0.002 | 0.753 / 0.004 | 0.838 / 0.001 | 0.790 / 0.002 | 0.542 / 0.048 | 0.629 / 0.014 | 1.000 | 0.489 / 0.072 | 0.814 / 0.001 | 0.756 / 0.004 | −0.615 / 0.018 | 0.816 / 0.001 |
| **Urea** | 0.694 / 0.006 | 0.362 / 0.183 | 0.628 / 0.014 | −0.522 / 0.055 | −0.535 / 0.050 | −0.474 / 0.079 | −0.810 / 0.001 | 0.367 / 0.184 | 0.513 / 0.058 | 0.646 / 0.012 | 0.710 / 0.007 | 0.710 / 0.007 | 0.337 / 0.218 | 0.634 / 0.013 | 0.489 / 0.072 | 1.000 | 0.721 / 0.005 | 0.769 / 0.004 | 0.008 / 0.976 | 0.687 / 0.009 |
| **Glucose** | 0.833 / 0.001 | 0.515 / 0.058 | 0.775 / 0.001 | −0.502 / 0.065 | −0.709 / 0.005 | −0.394 / 0.159 | −0.706 / 0.007 | 0.589 / 0.034 | 0.760 / 0.003 | 0.807 / 0.002 | 0.835 / 0.001 | 0.850 / 0.001 | 0.483 / 0.073 | 0.794 / 0.002 | 0.814 / 0.001 | 0.721 / 0.005 | 1.000 | 0.788 / 0.002 | −0.412 / 0.141 | 0.854 / 0.001 |
| **Triglycerides** | 0.920 / 0.001 | 0.486 / 0.071 | 0.832 / 0.001 | −0.439 / 0.109 | −0.448 / 0.104 | −0.442 / 0.113 | −0.712 / 0.006 | 0.541 / 0.048 | 0.792 / 0.002 | 0.638 / 0.013 | 0.797 / 0.002 | 0.832 / 0.001 | 0.517 / 0.057 | 0.764 / 0.003 | 0.756 / 0.004 | 0.769 / 0.004 | 0.788 / 0.002 | 1.000 | −0.266 / 0.329 | 0.853 / 0.001 |
| **HDL** | −0.229 / 0.414 | −0.329 / 0.235 | −0.355 / 0.197 | 0.523 / 0.054 | 0.570 / 0.034 | 0.029 / 0.914 | 0.282 / 0.310 | −0.400 / 0.147 | −0.516 / 0.057 | −0.294 / 0.289 | −0.507 / 0.062 | −0.523 / 0.054 | −0.502 / 0.065 | −0.380 / 0.166 | −0.615 / 0.018 | 0.008 / 0.976 | −0.412 / 0.141 | −0.266 / 0.329 | 1.000 | −0.407 / 0.142 |
| **LDL** | 0.907 / 0.001 | 0.520 / 0.056 | 0.866 / 0.001 | −0.485 / 0.073 | −0.668 / 0.010 | −0.405 / 0.143 | −0.635 / 0.013 | 0.601 / 0.031 | 0.760 / 0.003 | 0.784 / 0.002 | 0.806 / 0.002 | 0.913 / 0.001 | 0.410 / 0.145 | 0.742 / 0.004 | 0.816 / 0.001 | 0.687 / 0.009 | 0.854 / 0.001 | 0.853 / 0.001 | −0.407 / 0.142 | 1.000 |

# **Additional Table S6: Effect Size Summary for Key Experimental Outcomes (16‑week time point)**

| **Comparison** | **Outcome Variable** | **Mean Difference** | **Cohen’s d (95% CI)** | **p‑value** |
| --- | --- | --- | --- | --- |
| **AOM/DSS vs. Control** | IL‑6 (pg/mg protein) | +221.7 pg/mg | 3.15 (1.92, 4.38) | < 0.001 |
|  | IL‑1β (ng/L) | +70.2 ng/L | 2.94 (1.73, 4.15) | < 0.001 |
|  | miR‑122‑5p (fold‑change) | +2.81‑fold | 2.87 (1.59, 4.15) | < 0.001 |
|  | miR‑15b‑5p (fold‑change) | +2.44‑fold | 2.62 (1.38, 3.86) | < 0.001 |
|  | TNF‑α IHC area (%) | +28.5% | 3.41 (2.12, 4.70) | < 0.001 |
|  | Collagen area (%) | +24.3% | 2.88 (1.60, 4.16) | < 0.001 |
|  | Goblet cell count/HPF | −18.2 cells | −3.02 (−4.24, −1.80) | < 0.001 |
|  | Dysplasia score (0–3) | +2.4 points | 4.12 (2.75, 5.49) | < 0.001 |
| **AOM/DSS vs. *S. boulardii*** | IL‑6 (pg/mg protein) | −205.7 pg/mg | −2.41 (−3.57, −1.25) | < 0.001 |
|  | IL‑1β (ng/L) | −53.0 ng/L | −1.98 (−3.08, −0.88) | 0.002 |
|  | miR‑122‑5p (fold‑change) | −1.92‑fold | −1.94 (−3.06, −0.82) | 0.003 |
|  | miR‑202‑5p (fold‑change) | +1.65‑fold | 1.82 (0.72, 2.92) | 0.005 |
|  | TNF‑α IHC area (%) | −22.7% | −2.68 (−3.89, −1.47) | < 0.001 |
|  | Collagen area (%) | −18.1% | −2.14 (−3.28, −1.00) | 0.001 |
|  | Goblet cell count/HPF | +14.8 cells | 2.76 (1.56, 3.96) | < 0.001 |
|  | Dysplasia score (0–3) | −1.6 points | −2.84 (−4.01, −1.67) | < 0.001 |
| **AOM/DSS vs. *W. coagulans*** | IL‑6 (pg/mg protein) | −217.7 pg/mg | −2.38 (−3.53, −1.23) | < 0.001 |
|  | IL‑1β (ng/L) | −47.0 ng/L | −1.75 (−2.82, −0.68) | 0.006 |
|  | miR‑122‑5p (fold‑change) | −1.88‑fold | −1.90 (−3.02, −0.78) | 0.004 |
|  | miR‑202‑5p (fold‑change) | +1.70‑fold | 1.87 (0.76, 2.98) | 0.004 |
|  | TNF‑α IHC area (%) | −23.5% | −2.78 (−3.99, −1.57) | < 0.001 |
|  | Collagen area (%) | −19.3% | −2.28 (−3.44, −1.12) | < 0.001 |
|  | Goblet cell count/HPF | +15.3 cells | 2.85 (1.64, 4.06) | < 0.001 |
|  | Dysplasia score (0–3) | −1.7 points | −3.02 (−4.24, −1.80) | < 0.001 |
| ***S. boulardii* vs. Control** | IL‑6 (pg/mg protein) | +16.0 pg/mg | 0.23 (−0.77, 1.23) | 0.643 |
|  | IL‑1β (ng/L) | +17.2 ng/L | 0.64 (−0.37, 1.65) | 0.204 |
|  | miR‑122‑5p (fold‑change) | +0.89‑fold | 0.91 (−0.12, 1.94) | 0.081 |
|  | Dysplasia score (0–3) | +0.8 points | 1.42 (0.39, 2.45) | 0.010 |
| ***W. coagulans* vs. Control** | IL‑6 (pg/mg protein) | +4.0 pg/mg | 0.06 (−0.94, 1.06) | 0.901 |
|  | IL‑1β (ng/L) | +23.2 ng/L | 0.87 (−0.15, 1.89) | 0.089 |
|  | miR‑122‑5p (fold‑change) | +0.93‑fold | 0.94 (−0.09, 1.97) | 0.073 |
|  | Dysplasia score (0–3) | +0.7 points | 1.25 (0.24, 2.26) | 0.018 |
| ***S. boulardii vs. W. coagulans*** | IL‑6 (pg/mg protein) | +12.0 pg/mg | 0.17 (−0.83, 1.17) | 0.735 |
|  | IL‑1β (ng/L) | −6.0 ng/L | −0.22 (−1.22, 0.78) | 0.654 |
|  | miR‑21‑5p (fold‑change) | −0.51‑fold | −0.61 (−1.62, 0.40) | 0.223 |
|  | Dysplasia score (0–3) | +0.1 points | 0.18 (−0.82, 1.18) | 0.714 |

All effect sizes are reported as ***Cohen’s d*** (for parametric data) with 95% confidence intervals. Positive d values indicate higher levels in the first‑named group; negative d values indicate lower levels. ***Cohen’s d*** *interpretation: |d| ≈ 0.2 (small), ≈ 0.5 (medium), ≈ 0.8 (large).* ***Mean difference*** *units correspond to the original measurement units of each variable. CIs for Cohen’s d were calculated using Hedges’ g adjustment for small sample bias. All p‑values are from Tukey’s HSD post‑hoc tests following one‑way ANOVA (parametric) or Dunn’s test with Bonferroni correction (non‑parametric).*

**References**

1.	Sarver AL, Sarver AE, Yuan C, Subramanian S. OMCD: OncomiR Cancer Database. BMC Cancer [Internet]. 2018 Dec 6 [cited 2024 Jul 9];18(1):1–6. Available from: https://bmccancer.biomedcentral.com/articles/10.1186/s12885-018-5085-z

2.	Ludwig N, Leidinger P, Becker K, Backes C, Fehlmann T, Pallasch C, et al. Distribution of miRNA expression across human tissues. Nucleic Acids Res [Internet]. 2016 May 5 [cited 2024 Jul 9];44(8):3865–77. Available from: https://dx.doi.org/10.1093/nar/gkw116

3.	Yang Z, Wu L, Wang A, Tang W, Zhao Y, Zhao H, et al. dbDEMC 2.0: updated database of differentially expressed miRNAs in human cancers. Nucleic Acids Res [Internet]. 2017 Jan 1 [cited 2024 Jul 9];45(Database issue):D812. Available from: /pmc/articles/PMC5210560/

4.	Laganà A, Forte S, Giudice A, Arena MR, Puglisi PL, Giugno R, et al. miRò: a miRNA knowledge base. Database (Oxford) [Internet]. 2009 [cited 2024 Jul 9];2009. Available from: /pmc/articles/PMC2794794/

5.	Prabahar A, Natarajan J. ImmunemiR - A Database of Prioritized Immune miRNA Disease Associations and its Interactome. Microrna [Internet]. 2017 Feb 23 [cited 2024 Jul 9];6(1):71–8. Available from: https://pubmed.ncbi.nlm.nih.gov/28124611/

6.	Vlachos IS, Zagganas K, Paraskevopoulou MD, Georgakilas G, Karagkouni D, Vergoulis T, et al. DIANA-miRPath v3.0: deciphering microRNA function with experimental support. Nucleic Acids Res [Internet]. 2015 Jul 7 [cited 2024 Jul 9];43(Web Server issue):W460. Available from: /pmc/articles/PMC4489228/
